# Patients’ experiences of coping with Idiopathic Pulmonary Fibrosis and their recommendations for its clinical management

**DOI:** 10.1371/journal.pone.0197660

**Published:** 2018-05-23

**Authors:** Sameera Senanayake, Kim Harrison, Michael Lewis, Melitta McNarry, Joanne Hudson

**Affiliations:** 1 School of Sport and Exercise Sciences, College of Engineering, Swansea University, Swansea, United Kingdom; 2 Faculty of Allied Health Sciences, General Sir John Kotelawala Defence University, Rathmalana, Sri Lanka; 3 Department of Respiratory Medicine, Morriston Hospital, Swansea, United Kingdom; Universite de Bretagne Occidentale, FRANCE

## Abstract

**Background:**

Idiopathic Pulmonary Fibrosis (IPF) is a chronic, progressive and life-limiting condition. From a healthcare perspective it is vital to establish effective methods of improving the quality of remaining life in these patients. This requires a detailed understanding of the multiple impacts of an IPF diagnosis on the individual.

**Methods:**

We sought to understand how patients coped with their initial diagnosis, how they live with the disease day-to-day, and their experiences and opinions of the professional support they receive. A patient-centred approach was used to explore the social, psychological and physical impacts of IPF. Semi-structured interviews were conducted by an experienced academic. Interview questions were written by the researchers but guided by informal conversations with patients and clinicians. An inductive thematic approach was used to analyse the data, allowing us to identify common themes in the patients’ experiences.

**Results:**

Of fifty invited participants, ten took part in the study (aged 53–81 years; 9 male). Inductive analysis of interviews identified seven second-order themes and eleven first-order themes, represented by two General Dimensions: ‘*Patient experience with the condition’* and ‘*Patient-led recommendations for practice’*. The key message on ‘coping’ in these patients was that acceptance of their condition led to a sense of optimism. Participants reported using appraisal-focused coping strategies to change their perspectives (thinking positively) and emotion-focused strategies to overcome depression (the main opportunity for emotional expression being an IPF support group). The support group also facilitated problem-focused coping: individuals exchanged knowledge and experience and gave one another tips on how to live with their condition.

**Conclusions:**

Health professionals should provide patients with information that focuses on *living* with IPF, encouraging them to make lifestyle changes and adaptations to improve quality of life. Family members should receive education about IPF so that they can support such changes. Patients should be encouraged to join a support group and to participate in physical activity (again preferably group-based). This study offers novel findings that will help inform much-needed changes in the practice of supporting IPF patients to cope with their diagnosis and disease progression.

## Introduction

Idiopathic Pulmonary Fibrosis (IPF) is a chronic, progressive and life-limiting disease of the lungs. It’s cause is unknown but it is recognised as a distinct clinical form of the idiopathic interstitial pneumonias [1]. Progressive fibrosis of the alveolar walls causes impairment of gas exchange, decline in lung function and ultimately, death from respiratory failure. Consequently, patients with IPF present with symptoms of increasing breathlessness and fatigue whilst many also develop a dry cough, which can prove difficult to treat. In the UK, IPF has an annual incidence of 4.6 per 100,000 and this is increasing by 5% per annum, a trend mirrored by hospital admission rates [2]. Whilst new therapies that slow the decline in lung function have recently been recommended for IPF (Nintedanib and Pirfenidone) [1] there is still no cure. The median survival for patients diagnosed with IPF is 2.5 to 3.5 years although the range is wide [3], some following a slowly declining trajectory over many years whilst others may have a rapidly progressive course. Thus, individual patients are confronted with both the physical limitations imposed by the disease and the psychological impact of an ultimately fatal condition with uncertain rate of progression. Therefore, an important aspect of caring for people with IPF is to help preserve their Quality of Life (QoL), taking into account the impact thereon of physical, psychological and social wellbeing [4,5]. A recent review [6] highlighted studies that have attempted to measure or improve QoL in IPF (using patient-reported outcome measures and interventions) and those that have identified unmet patient needs (such as emotional support and information resources). A few studies have assessed the individual psychological impact of an IPF diagnosis and also patients’ experiences of living with the disease [7–11].

The aim of this study was to explore the idiographic experiences of patients with IPF within our local health board in South West Wales, and thus to improve our understanding of the psychosocial impact of the disease in this region. We used a qualitative interview approach that enabled patients to ‘tell their own stories in their own words’, so that they could focus on the issues and experiences that had personal meaning for them in four specific areas: how they coped after receiving the diagnosis; how they live with the disease day-to-day; their experiences and opinions of the professional support they received; and their views on the pulmonary rehabilitation programme they were offered. In so doing, we hoped to gain insight into how to best support the psychological needs of individuals diagnosed with IPF.

## Materials and methods

### Participants

The study was performed in accordance with the Declaration of Helsinki. Ethical approval to conduct the study was obtained from the School of Sport and Exercise Sciences Ethics Committee at Swansea University. Members of the research team attended a monthly support group for IPF patients held in a community setting in Swansea (South West Wales, UK), following which they approached patients to invite them to participate in the study. (The support group meetings aimed to provide an opportunity for IPF patients to meet in a social setting and, if they wished, to discuss their experiences of the disease and thus to support one another—for example regarding anxieties and coping with the challenges of daily living.) Patients who agreed to participate were then contacted individually by letter to arrange the interviews, which were conducted at Swansea University. All recruited individuals provided their written informed consent to participate in the study. Our purposive sampling criteria required that participants had been diagnosed with IPF, were aged 18 years or older and were able to write and converse in English (to facilitate data collection). Given the individuality of the disease, we contacted a large pool of patients (50 individuals) to recruit a variation sample that would allow us to capture a wide range of perspectives from participants.

### Method

Participants attended semi-structured interviews conducted by two of the authors (SS, JH) following monthly support group meetings held in Swansea, South West Wales. Participants attended semi-structured interviews conducted by two members of the researcher team. Interviews lasted 45–60 minutes and were audio recorded and transcribed verbatim. An inductive thematic approach [12] was used to analyse the transcribed data: raw data themes were recorded during the interviews and these were tracked and continually revised throughout the transcription process. Details of the interview procedure (including example interview questions) and the data analysis methods used are provided in S1 File.

## Results

A total of 50 IPF patients registered in the support group were contacted by post and 10 volunteered to take part in the study. No replies were obtained from the other 40 individuals, and no explanations were sought from those who did not respond (in line with our ethical approval). After analysing the transcripts from these ten participants the research team considered that data saturation had been reached and so no additional patients were invited to participate. The study participants ranged in age from 53 to 81 years (mean = 70.5 ± 10.4 years), one of whom was female. Participants reported durations of disease onset ranging from 7 months to 10 years: onset under one year–five patients; onset 3–5 years (two patients); onset 8–10 years–three patients. Only one participant used oxygen during the interview, while two participants reported using oxygen during strenuous activities in daily life. All participants were Caucasian. Eight participants attended the interview on their own (one of whom was interviewed at home) and two attended with their spouses. In these latter interviews, the participant’s spouse contributed to the discussion, simultaneously offering verification of the participant’s responses, but also often encouraging further reflection or interpretation from the participant.

**Fig 1:**
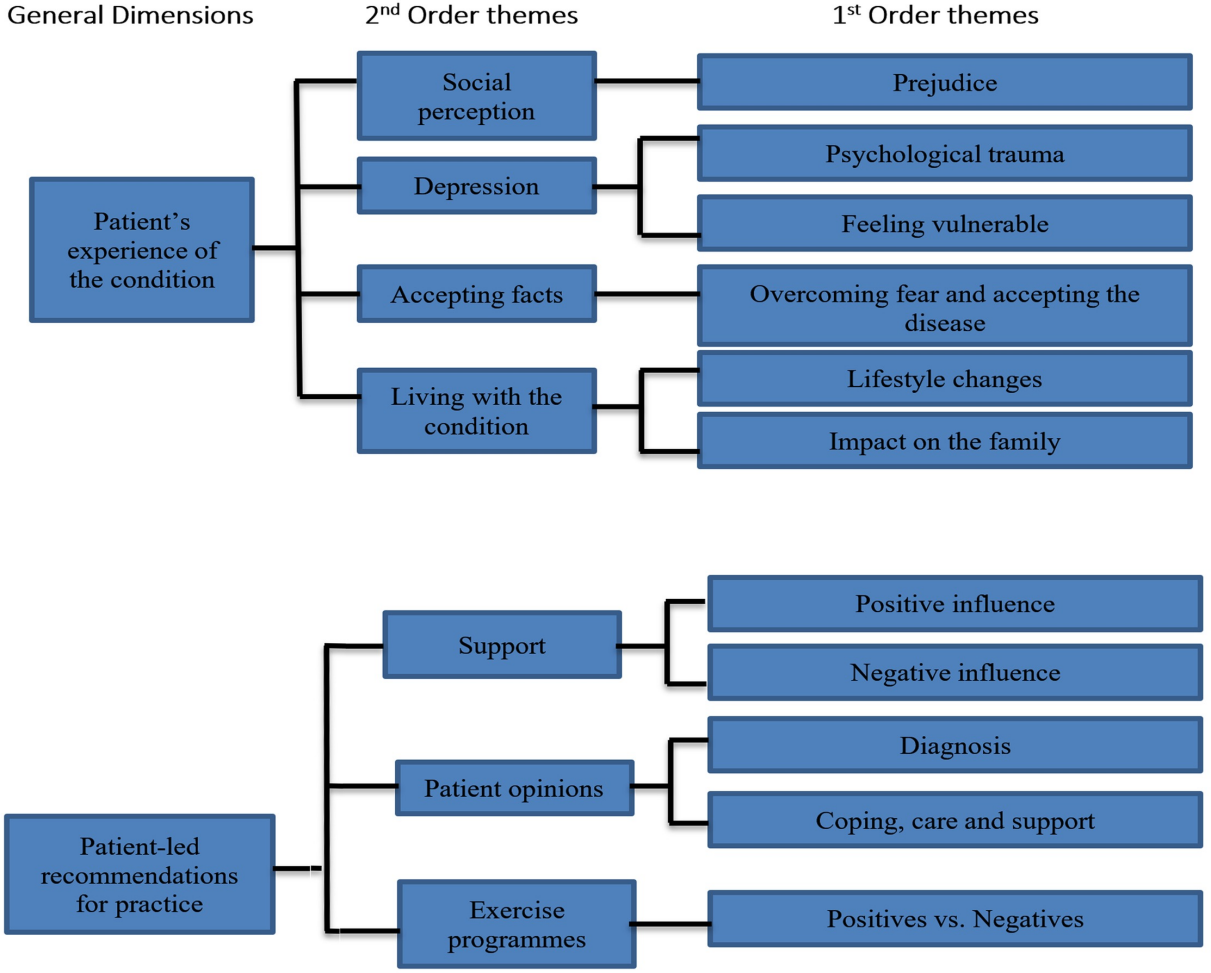
Key themes representing patients’ experiences of IPF and patient-led recommendations for practice.

Inductive analysis of the patient interviews led to identification of seven second order themes and eleven first order themes that are represented by two General Dimensions: *Patient experience with the condition* and *Patient-led recommendations for practice*. An overview of these themes and their hierarchical relationships with each other is shown in Fig 1.

### General Dimension 1: Patients’ experiences of IPF

This General Dimension was underpinned by 4 second order themes (*Social perception*, *Depression*, *Accepting facts*, and, *Living with the condition)* that mainly describe the psychosocial impact of the disease on the patient, how it has affected his or her life, and the impact of IPF on the patient’s family members. These second order themes were derived from (and reflect) both negative and positive responses from the participants, and were categorised collectively as they relate to the same shared theme. The theme explanations below illustrate the positive and negative responses provided by participants in relation to each theme.

#### Social perception

In this context, ‘social perception’ describes an individual’s experiences of negotiating the social implications of IPF, including their feelings about how other people perceive and treat them and how this impacts on their psychological well-being. Across all the interviews, it was evident that participants and their close family members had little or no knowledge of IPF before diagnosis, and most had not even heard the phrase IPF previously. This lack of public awareness could be due to the limited availability of information about the disease: *“A lot of people don’t know what it is*. *They understand lung cancer but when it comes to IPF they are like ‘what is that*?*’*” (Patient J). This can lead to perceived prejudice: *“When people see you coughing they say ‘take cough medicine*, *take this*, *take that’*, *they don’t understand it”* (Patient B) and *“they would say*, *‘why you are panting when you are doing nothing literally*?*’”* (Patient B). When patients face these kinds of responses on a daily basis they feel isolated and somewhat stigmatised, which appeared to be contributing factors to the onset of depressive symptoms in some patients.

#### Depression

Most of the patients interviewed displayed symptoms consistent with depression or low mood. This is likely attributable to the psychological trauma associated with their disease, as noted by one participant who commented on what their diagnosis meant to them: *“Death*. *Certain*, *definite death*.*”* (Patient H). Aside from feelings of depression a sense of vulnerability was also apparent among participants, adding to the feelings of depression, and this appeared to stem from their lack of knowledge about the disease. The comments of two participants illustrate this: *“Lack of information always causes more worries”* (Patient A) and *“Well*, *I’m in the dark about what you can do to help the condition”* (Patient B). Inaccurate or misinterpreted information about the nature and prognostic implications of IPF appears to be a common problem, as reflected in the perceptions: *“If I was diagnosed with lung cancer it would have knocked me over*, *but knowing I have a fighting chance as things are*, *it’s not bothering me”* (Patient E), and *“I was pleased it wasn’t cancer”* (Patient H).

The psychological trauma caused by the disease also appears to derive from altered perceptions of self: *“I feel like a battery*, *your energy levels go down and you would have to stop”* (Patient J). When patients could not live up to their self-expectations and their previous self-perceptions of ability, they experienced feelings of self-pity that further fuelled their anxieties about the debilitating nature of the disease. As one participant commented: *“Sometimes I feel ashamed about the things I can’t do”* (Patient A). Another participant explained: *“If I go out I have to plan a route*, *that I know which is flat*. *So that I know*, *if I got to that point I can get back*. *You have to think ‘if I can’t come back who am I gonna call to come pick me up*?*’*. *You know*, *things like that*. *It really takes it out of you*, *it’s surprising to see how much it takes out of you*. *After being there and doing something I feel like an old man of 90*.*”* (Patient G, age 53). Furthermore, these IPF patients often experienced regret and frustration mainly due to the morbidity of the disease and because they were being prevented from living their latter years as planned: *“I do get frustrated when it physically affects me*, *not being able to do things that I planned to do”* (Patient J) and *“You plan to do all these things that you wanted to do all these years*. *But here we are*.*”* (Patient I).

#### Accepting facts

‘Accepting facts’ refers to the stage at which all participants indicated they had started to come to terms with the condition and attempted to move on with their lives. As the quotes below illustrate, usually this began with accepting the fact that they have the disease and it ended with the sense of achievement gained from being able to cope with (and adapt to) living with the disease. When accepting the facts about, and the consequences of, the disease, most participants were initially optimistic: *“I was determined to live my life to the fullest”* (Patient A) and *“I told myself ‘you have to try anything and everything’”* (Patient C).

This positivity and optimism was enhanced further when individuals felt that their lives mattered, thereby nurturing their sense of self-worth. This derived in part from knowing that there are people and groups in society who care about them: *“I’ve had a leaflet from the lung association”* (Patient B). Once these participants had achieved acceptance of the disease, they were able to adapt to the disease accordingly: *“I learnt a few things like when it comes to climbing steps it’s easier to breathe in when you take off the step and breath out when you land on the step*” (Patient G) and *“If I am going out I need to plan a route which I know is flat”* (Patient G).

Following on from this acceptance and adjustment, some participants attempted to engage in physical activities, the successful completion of which gave them a sense of achievement. This was cause for renewed optimism, and an increased commitment to self-care as they looked to the future with hope: *“It was keeping my lungs clear (now I have a lot of mucus and phlegm) and it felt better*. *When I was done*, *you felt that you have achieved something”* (Patient F). Hearing participants’ experiences, it was evident that overcoming fear of the disease and accepting the facts about it represented the initial stage of moving towards an elevated quality of life; individuals had a sense of starting to live again, despite living *with* the condition, and of achieving some normality to their day-to-day lives. Once they had successfully navigated through this life experience, patients were often able to ‘move on’, overcoming the psychological trauma caused by the diagnosis and associated morbidity of the disease.

#### Living with the condition

These themes describe how the disease affected the daily lives of the participants and the adaptations they had made to cope with the condition on a daily basis. Overwhelmingly, IPF had a huge impact on the participants’ lifestyles: *“I used to play a lot of golf*, *swimming*, *cricket*, *you name it*. *I’ve had no problem*, *but now I’m a television viewer”* (Patient B) and *“I’ve sometimes found lifting whatever across the garden these days is difficult*, *but before it was easy”* (Patient E).

Most of the interviewees had to change their lifestyles completely following diagnosis, adjusting to living with the condition through planning and prioritizing activities. They mentioned *“finding the simplest way to do things”* and *“I try to keep everything in precise order*, *so things at the same place all the time”* (Patient A) and *“It’s sort of reorganizing your life*, *try and do everything in one place before you move to another”* (Patient B). Such modifications helped significantly in overcoming the obstacles they faced due to the debilitating nature of the disease. Participants’ comments showed that the disease also affected their family members: *“Then my daughter read it and she was badly distressed and she kept it from me*. *When it’s sitting there in front of you*, *you think OMG* [Oh My God]*”* Patient H) and *“My son had a shock when he saw me with the oxygen”* (Patient F). Interestingly, the participants did not discuss coping strategies employed by their family members but some did mention protective behaviour in both directions. Some participants mentioned protecting their family members, and others noted that family members protected them, from some of the stark realities of the disease: “*My daughter knows*, *my husband knows*. *He has been very protective in a loving way*. *My son*, *haven’t told him*” (Patient H).

### General Dimension 2: Patient-led recommendations for practice

The second general dimension was made by three second order themes (support, patient opinions and, exercise programme) which mainly describes patients opinion and thoughts on how to minimise the impact of IPF on daily life and the positives and negatives of support available for IPF patients. As above the theme explanations illustrates both the positive and negative responses given by the participants in relation to each theme.

#### Immediate responses to diagnosis

Receiving initial information about IPF at the point of diagnosis was a traumatic experience for participants. Most had never heard of the condition previously and the information presented a stark and upsetting prognosis: *“It came as a little bit of a shock*. *It affected me”* (Patient I) and *“Very shocked*. *Really upset at the time*. *Coming to terms with it slowly”* (Patient H). Two participants commented about the way in which they were told about the disease: *“They gave me a booklet and I wish I didn’t read it”* (Patient H) and *“To give it to someone as soon as they diagnosed it*, *I think it was not a good idea”* (Patient G). The initial shock and unexpected diagnosis of a disease about which they were unaware left many patients looking for someone to blame, as they felt somehow victimised by their diagnosis. However, they were unable to attribute this blame to someone or something, which only added to the immense frustration and psychological trauma caused by their diagnosis: *“I feel picked on*, *I feel I haven’t smoked for all these years*, *I don’t drink*, *I’m not overly fat*, *why is this happening to me*?*”* (Patient G).

Each of the study participants had an individual perspective on how to cope with the disease and suggestions on how to improve the care and support available for IPF patients. Overwhelmingly, one of the key factors identified was the lack of available and appropriate information on the disease. Both ignorance and ambiguity about the disease contributed to the trauma that they were already experiencing with their diagnosis: *“They say you are like this and that’s how you are going to end up like*. *And you think*, *what’s going to happen in between*?*”* (Patient G). Participants offered a number of suggestions on how to address this, including involving patients in developing materials for other sufferers and giving patients a role in educating health professionals, to help them to understand the patient’s experience of diagnosis and the disease trajectory. One participant suggested that *“Initially I will direct them to our support group*. *Then I would ask someone who had experience of the condition and the rehabilitation program to talk about rehab* [sic], *who can pass on their true feelings—not a clinical thing*, *somebody talking from the heart who had done it”* (Patient D). Another commented “*I think you need it in age groups*, *which would help”* (Patient G), and another felt that *“You should find out all the facts*, *face up to the facts*, *accept what is inevitable and live accordingly”* (Patient A). They felt that these strategies would help future IPF patients to appropriately fill the information gap they had experienced when they were diagnosed with the disease.

#### Support

Participants’ comments indicated that support from others could be perceived as either a positive or a negative influence. Most of the participants experienced positive support after their diagnosis with IPF, from either family or peers and not surprisingly this was viewed as very important in helping them to accept the disease and to overcome the depression they experienced: *“They would do anything if I ask my family”* (Patient A). The peer support they experienced mainly came from the monthly support group that these participants attended and, as was the case with family support, support from others with the disease was perceived as vital in overcoming feelings of depression and adapting to living with the condition: *“The positive is that I’ve met people similar*, *some of them are very nice’* (Patient A) and *“There was company*, *people with the same disease*. *I found I was looking forward to going*. *And I thought it was doing me good”* (Patient H). Others discussed the informal educational role this peer support played: *“We have a cup of tea*, *cake and we talk about how things are*. *They get people from different ways of life to talk about the problem”* (Patient F) and *“Just having a chat about families and how they are coping*, *you know that’s the biggest part of it*. *Getting together and talking to people with the same problem’* (Patient F). Some found comfort in just seeing other people who are in a worse condition than them: “…*but it was nice to see there were people worse than I was*” (Patient G).

Although the support group was an incredibly powerful coping mechanism for the participants, its influence was not consistently positive: “I couldn’t help thinking that this is like Shipman’s waiting room^*^, *waiting for each one to pop off you know*. *Because there is always news that somebody is gone and that’s a person you know and I think*, *my name would be there one day wouldn’t it*?*”* (Patient A) [^*^Dr Harold Shipman was a notorious General Practitioner in the UK who was convicted of murdering (via euthanasia) several of his elderly patients]. This patient also discussed the impact of a shared common fate: *“…down turn because you all know that you are going sooner than you’d like to be”*.

Family support was perceived in a similar vein: overall it was received positively but this was not always the case. For example, a number of respondents discussed that family members contributed to their lack of independence by making decisions about their lifestyles for them, or by doing things for them that (with some effort) they were still able to do for themselves. One participant commented *“I got told off by my daughter for cutting the grass*. *But I got to do it*. *I got to do it when I get the chance”* (Patient F), whilst another noted *“Eventually she is talking about us moving downstairs and staying downstairs once it gets to a certain point—well I don’t want that*. *I’d rather take 10 minutes to go upstairs to go to bed than staying downstairs all the time”* (Patient G).

#### Exercise programmes

Study participants felt that physical exercise could benefit them physically and could positively influence their mental well-being, helping them to deal with their feelings of depression and to simply carry on with life. They discussed the benefits they had experienced from exercise: *“Mentally I feel good”* (Patient D), *“I find it useful for circulation on the legs”* (Patient I), *“I’m sleeping better on the days I was doing it”* (Patient G) and *“I found after exercising I slept better*, *woke up better and it improved my flexibility*. *Mentally you are feeling better too and it helps”* (Patient I). However, participants’ experiences of exercise did not always remain positive over time owing to the high oxygen demand of exercise and the negative effects of this for some patients: *“I found it a struggle”* (Patient C) and *“I found it was a struggle*, *because I’m out of breath when I do it”* (Patient D).

Patients’ comments indicated that they preferred group activities when it came to exercise programmes, for example: *“It was about meeting others too*” (Patient C) and “*You met people and after that you got a cup of tea*, *sat down and had a chat*. *It was nice*. *But you won’t get that in a gym*” (Patient G) and “*There was company*, *people with the same disease*. *I found I was looking forward to going*. *And I thought it was doing me good*” (Patient H).

## Discussion

Idiopathic pulmonary fibrosis (IPF) is a life-limiting disease that has no curative options [1] and this condition is little-recognised and poorly-understood by the general public. From a patient’s perspective a diagnosis of IPF is therefore often completely unexpected and confusing, provoking a range of psychological responses that cause much anxiety. A better appreciation of patients’ personal experiences of being diagnosed (and then coping) with IPF is critical to changing the way we educate and support newly-diagnosed patients about their condition. Our exploration of individual experiences of IPF offers insight into the psychosocial implications of IPF, its impacts on psychological and physical well-being, and the physical and psychological coping strategies that patients adopt to cope with these. This has enabled us to present here some patient-led recommendations for healthcare workers to support IPF patients from the point of diagnosis and throughout the progression of their disease. We analysed our data inductively to allow themes to be generated from the data rather than using a deductive theory-driven approach [12]–further discussion of this approach is provided in S2 File.

### Patients’ experiences of IPF

#### IPF and need-thwarting

Our participants told us that they had experienced insensitive and unsympathetic comments regarding their condition and the struggles they faced each day, leading them to feel less related to (and more isolated from) others in wider society (reduced relatedness satisfaction). The majority of participants said that, because of this, they had limited their social interactions and confined themselves to a ‘safe space’ among family, close friends and sometimes the weekly IPF support group. Russel et al. claimed that a poor understanding of IPF amongst the general public contributes to the emotional impact of IPF on patients (depression, fear, frustration and isolation), causing them to withdraw from social relationships [11]. This reduced social interaction results in an unfulfilled need for relatedness (need-thwarting) in these individuals and negatively influences their overall well-being and quality of life. Schoenheit et al. made similar observations, claiming that IPF patients express difficulties in maintaining relationships owing to others’ lack of awareness and understanding of the disease [8]. Sampson et al. observed that deterioration in health caused patients to restrict their activities (by confining them to indoors) rather than seeking assistance to maximise their functional activities (through socialising and conducting outdoor activities), thereby increasing the risk of social isolation and leading to diminished possibilities [9]. Similarly Swigris et al. reported that some patients avoided engaging with crowds of people simply because of the fear of catching a respiratory disease that could lead to their demise [13]. Bonella et al. suggested that awareness of IPF among the general public should be increased through campaigns emphasising the chronic and debilitating nature of the disease [14]. Our participants’ responses were consistent with depressed mood, indicating that their sense of incompetence, lack of autonomy and lack of intrinsic motivation were having a substantial negative impact on their well-being.

Not surprisingly, given its physically-restrictive nature, IPF had a substantial impact on patients’ satisfaction regarding the need to feel competent. This impact was especially apparent in the execution of what were previously simple, taken-for-granted daily activities. The patients we interviewed often experienced signs of breathlessness and fatigue during the simplest of physical activities like walking or moving an object from one place to another. In addition, most of our participants had previously been involved in sport or other recreational activities such as walking prior to the onset of IPF, and no longer being able to participate in these activities further thwarted their need to feel competent. This need-thwarting leads to frustration and was one of the most common observations we made during our interviews, along with a sense of shame and self-pity that stemmed from the morbidity impact of IPF. Patients’ reduced competence satisfaction was fuelled by the thwarting of their need for autonomy as most had to seek help in carrying out the activities of daily living. Often family members stepped in to help these patients even before their help was solicited; whilst participants appreciated their help, family members were unknowingly undermining the individual’s sense of autonomy and thus their competence satisfaction. Previous studies have similarly reported that patients’ inability to perform routine daily tasks negatively impacts on their emotional wellbeing [8,13]. Patients interviewed by Swigris et al. commented that the most detrimental aspect of this disease is the fact that they are less independent than previously, leading to a loss of privacy because they needed help from others [13].

A lack of understanding about the disease compounds this situation. Patients are reluctant to participate in physical activity because they are afraid of its possible impact on their condition, further reducing autonomy and competence-need satisfaction. Carers are often unsure how to help patients and express concerns regarding the monitoring of breathing, coughing and the use of oxygen [9]. Sampson et al. reported that patient-clinician consultations are mostly disconnected from the demands of practical day-to-day life. Patients often feel that they cannot interpret disease-focused assessments (like lung function tests) in relation to predicted future exercise capacity, causing them to simply avoid physical activity (presumably as a conservative approach to avoiding exacerbation) [9]. Cicutto et al. [15] also found similar attitudes to exercise in COPD patients, as did Fernandez et al. [16] in cancer patients, illustrating that this is a common observation in the literature relating to many chronic diseases.

#### Coping mechanisms and re-establishing need-satisfaction

There was evidence that our participants had also developed coping mechanisms that employed emotion-, problem-, appraisal-, approach- or support-focused strategies that helped them restore need-satisfaction after diagnosis. It was apparent that for many individuals the key to coping with IPF was an acceptance of the reality of their condition, as this subsequently led to a sense of optimism.

During the early stages of diagnosis (following initial emotional upheaval) participants used appraisal-focused coping strategies to purposely change their perspectives–this allowed them to start thinking more positively and to gain some determination to live the remainder of life to the fullest. One such example of this positive thinking and optimism was seen in a patient who had bought football tickets for the following season whilst knowing that his condition meant he had a relatively low likelihood of living to watch the games. Others have also observed that patients and family members try to maintain a normal life for as long as possible by living in the moment, while acknowledging that this is difficult as the disease progresses [10].

Participants used emotion-focused coping to overcome depression and to face the reality of their condition, mainly through the opportunities for emotional expression that the IPF support group offered. As Folkman and Lazarus [17] suggested, emotion-focused coping was employed alongside social support from others who shared their experience in the support group. The simultaneous use of emotion-focused coping and social support was also evident in the participants’ discussions of how family and friends had helped them to overcome the initial trauma and fear of diagnosis. Importantly, their recognition of the support and care from others helped to restore both lost self-worth and relatedness-satisfaction. The support group also functioned to facilitate problem-focused coping as individuals could exchange knowledge and experiences about the disease and provide tips on how to cope with the condition, which they would be unlikely to receive from a medical professional. There are however some negative associations with support groups. For example, Lindell et al. observed that health-related quality of life was diminished after patients had attended a six-week support group (although other benefits were reported, including reduced feelings of isolation in patients and reduced stress in their care partners)[7]. Sampson et al. suggested that seeing people in support groups who are further ahead in terms of disease progression threatens the ability of some patients to cope with the condition [9].

Participants’ effective employment of support-, appraisal- and emotion-focused strategies for coping with the wider psychological and personal implications of IPF meant that they were then able to use problem-focused coping to deal with daily challenges. They did so by adapting to their condition using small adjustments to the ways in which they conducted previously taken-for-granted activities. Sampson et al. stated that patients use a day-by-day coping strategy to adapt to their condition [9]. Swigris et al. observed that the majority of patients in their study were forced to plan everything ahead, leading them to analyze every activity before starting it [13]. Being able to accommodate the challenges IPF presents to once again conduct activities of daily living, such as going for a walk, provided a sense of achievement and led to a regained sense of competence and enhanced well-being. Some of our patients found comfort in a somewhat unorthodox manner by feeling good about themselves after seeing patients who were in a much worse condition than themselves.

### Patient-led recommendations for need-satisfaction

#### Information

Lack of information is one of the main issues that IPF patients face. This led many participants to feel uncertain about what they could and could not do after diagnosis. They felt uninformed and unable to make accurate judgements of their competence to carry out physical activities, resulting in the adoption of a sedentary lifestyle. The information given to these patients on IPF at the point of diagnosis was presented in an information booklet including general information on the disease, which emphasised life expectancy and what the patient could expect to happen towards the end of their life. However, it offered little information on what patients might expect to experience in the shorter term or how they might best cope with the condition. This often caused shock, anxiety and trauma, and subsequently led to depression-like symptoms in these patients. It should be noted that our patient were perhaps advantaged in receiving such information: patients have previously commented that they do not receive sufficient information on the practical management of the disease (ranging from advice on oxygen therapy, nutrition, exercise, the management of cough, managing breathlessness and, most importantly, how to cope with the disease towards the end of life) [8,9,11,14]. Others have noted that learning about the fatality of the disease is overwhelming for patients, and that they often find the amount of information they received overpowering; these patients instead prefer to be given information about the disease gradually [10]. In fact Bonella et al. [14] highlighted ‘improved information sources’ as one of the five unmet needs in IPF care after consulting eleven European patient advocacy groups (an initiative that led to the development of the European IPF Patients’ Charter). Based on our participants’ stories we recommend that the information given by health professionals should focus on living with IPF, maintaining quality of life through lifestyle changes (planning and prioritising) and encouraging patients to remain as physically active as possible. This would help patients to maintain feelings of competence and autonomy, and would encourage a proactive coping approach in patients from the point of diagnosis.

#### Support for need satisfaction

Family support plays a key role in maintaining the well-being of IPF patients, so it would be beneficial if immediate family members also received education about IPF and its effects on the lives of both the patient and themselves. Russel et al found that caregivers were inadequately prepared for the role of the caregiver owing to a lack of information about the disease and the absence of psychological support, resulting in an inability to relate to the patient [11]. Sampson et al. recommended that the role of the carer should change from ‘passive observer’ to ‘active member’ throughout the patient’s healthcare pathway, and that they should have access to better advice and support to fulfil their role in managing the domestic environment for patients [9]. This is an important aspect of providing appropriate social support for patients, especially in relation to being physically active. We suggest that an emphasis should be placed on how *not* to support these patients; for example, immediate intervention to help patients when performing physical activity or activities of daily living could thwart autonomy and competence satisfaction and lead to feelings of shame and subsequently depression. Instead, family members could be encouraged to help patients employ problem-based coping strategies, making lifestyle changes to enable continuation of activities that they performed prior to diagnosis. Furthermore, it is highly recommended that patients join an IPF-specific support group if such a facility is available. This will help them fulfil their needs for relatedness, while allowing them to obtain valuable knowledge about their condition from fellow patients who are experiencing different stages of the disease. Importantly, this will help increase patients’ awareness of what to expect in the future, and peer advice will assist them in employing proactive-coping strategies, with a view to maintaining competence and autonomy need satisfaction. Comments from participants in this study indicate that in general patients would benefit from participation in physical activities, especially in a group setting. Such benefits might include improvements in physical or psychological well-being (perhaps helping to restore competence satisfaction or to reduce feelings of social isolation), although further studies are needed to provide evidence of this.

### Limitations

Of the 50 patients approached 40 did not respond and the nature of our recruitment process meant that we had no follow-up information about the reasons for lack of replies. Although our sample was therefore restricted to ten participants we believe that data saturation had been reached at this level so we are confident that our results are robust. The demographic of the participants was quite focused and this might bias our results to a degree: 1) Participants were all resident in Swansea (South West Wales) or its immediate surrounding areas; 2) Participants were recruited from a single IPF support group which was part of the palliative care pathway of the local health board. This type of support system might not be generally available across other health boards in the UK or in other countries, so our findings (especially those relating to opinions on the support group) will be biased to this demographic; 3) The stage of disease progression and the time since diagnosis for our patients ranged from 1–10 years and, although this range is wide, the relatively small number of participants means there was some clustering into early/late stages of disease progression. Recently-diagnosed participants are likely to have different opinions about the disease and its impact on their lives compared to those who have been living with the disease for much longer. For each of these reasons we must be guarded in our interpretation of the results and in any attempt to generalise the findings to wider populations.

## Conclusions

Our study evaluated the experiences of local patients with IPF regarding their condition. It offers novel findings that will help to inform much-needed changes in the practice of supporting IPF patients to cope with their diagnosis and disease progression. By interpreting these experiences within the theoretical frameworks offered by self-determination theory and the transactional model of stress and coping, we add insight into the mechanisms that underpin the impact of IPF on psychological well-being. Furthermore, our recommendations are strengthened by their grounding in both theory and practice, and by ensuring that we considered the varying personal experiences that are characteristic of living with IPF. Based on our findings, future support for patients with IPF would benefit from a clear focus on providing effective and considerate communication about the disease. In addition, patients and their families should be better informed about the potential benefits of attending peer support groups, and about the types of coping strategies employed by their peers to ‘live better’ with their condition.

## Supporting information

S1 File Details of the interview procedure and the data analysis methods used. (Table A) Example interview questions.
(DOCX)

S2 File Discussion. Interpretive frameworks.
(DOCX)

S3 File Transcripts of participant interviews. (PDF)
