# Supplementary material for: Patients’ experiences of coping with Idiopathic Pulmonary Fibrosis and their recommendations for its clinical management
Source: PLoS One. 2018 May 23;13(5):e0197660. doi: 10.1371/journal.pone.0197660 (PMC5965862; doi:10.1371/journal.pone.0197660)
Supplement: S1 File — (Table A) Example interview questions. (DOCX) [file pone.0197660.s001.docx]

**Method: Interview Procedure**

Participants attended semi-structured interviews conducted by an experienced senior academic at Swansea University (JH, female, PhD) with extensive experience of conducting qualitative research, but with little knowledge of IPF prior to conducting the interviews (thus helping to reduce interviewer bias). This interviewer ensured that patients were put at ease during the interviews and was skilled in facilitating discussion of patients’ personal experiences of their disease. A second academic was also present at the interviews (SS, male); this researcher was knowledgeable about IPF and so led specific questioning about patients’ experiences of exercise rehabilitation and its role in disease management. The semi-structured interviews followed the steps outlined by Rubin and Rubin [1], starting with introductory questions then transition questions, prior to posing the main and summary questions. A definitive list of the interview questions cannot be provided as these were led by the participants’ responses, but example questions and their intended focus are provided in Table 1. The research team devised these questions and was guided by informal conversations (during the initial support group visit) with both patients and clinical staff involved in IPF care by SS and MAM.

**S1 File (Table A): Example interview questions**

| **Question type** | **Example question** | **Purpose of the question** |
| --- | --- | --- |
| Introduction | Can you tell me about the onset of your condition and a little bit about it? | This question aimed to engage the patient with the topic and to allow them to express their personal understanding of their condition. |
| Transition | How does IPF make you feel on a day-to-day basis? | This question was used to gain an overall impression of the psychological impact of the disease from the patient’s perspective, and to link the introductory and main questions. |
| Transition | How do you make the most of it? | These questions helped to facilitate the flow between topics of interest during the interview. These types of question were followed by a main question, depending on the patient’s response. |
| Main | If you think back to your life before being diagnosed with your medical condition, what are the things that you did before but are prevented from doing now? | This type of question was asked to get an indication of the patient’s lifestyle before diagnosis of the disease and how IPF had affected the patient’s daily life. This is the key information in which the interviewers were interested. |
| Summary | Is there any other way that it affects you? | These questions were intended to sum up the main topics of the interview, allowing the patient to summarise their overall experiences and personal perspectives of living with the disease. |

Interviews lasted between 45 and 60 minutes and were audio recorded and transcribed verbatim. After the first interview was transcribed, the interviewers reflected on the interview and the responses yielded; this resulted in minor changes to the wording of opening questions to improve their focus. To maintain anonymity, pseudonyms were given to individual participants when reporting their responses and all names and locations were redacted from the transcripts.

**Data Analysis**

Although several qualitative studies have been conducted on patients’ experiences with IPF most of these have focused on the impact of specific aspects of the disease on quality of life. Our study sought to discuss the overall physical, social and psychological impacts of IPF on daily life so we employed an inductive thematic approach [2] to analyse the data. This approach provides flexibility and the ability to use multiple theories to process the data [3], helping us to better understand patients’ individual stories and to more effectively identify common themes amongst the patient group. Using this approach, notes and initial impressions (raw data themes) were recorded during the interviews and these ideas were tracked and continually revised throughout the transcription process. This analysis followed strategies outlined by Miles and Huberman [4] where quotes (representing raw data) were highlighted and labelled with an initial raw data theme that reflected the idea or meaning conveyed in the quote. This process continued until all quotes relevant to the research aim were highlighted and either allocated to an existing raw data theme or, if they did not fit into an existing theme, given a new raw data theme label. To ensure that the original meaning of the raw data was retained, following this initial process, the primary researcher (SS) returned to the transcripts to verify the allocations of labels to the raw data. The raw data generated by interview quotes were coded using patient identification codes to allow us to view how representative each theme was of overall participants’ experiences. After the data were coded and categorized into raw data themes, they were reviewed systematically to identify common meaning across different raw data themes. Those that had shared common meaning were grouped together to form a first order theme, which was given an appropriate label to represent the shared common meaning of its composite raw data themes. This process was repeated with the resulting first order themes to produce second order themes, and finally with the second order themes, to produce general dimensions. Each level of analysis and categorisation led to increasingly more abstract categorisation of the concrete raw data quotes. Hence the trustworthiness process described below was essential in ensuring the final higher order themes maintained the integrity of the original data and continued to reflect the participants’ experiences.

An extended process of peer debriefing was used to establish the trustworthiness of the data analysis where (JH) acted as a critical friend to challenge and check the credibility of the data analysis and interpretation. The critical friend independently reviewed each transcript and highlighted salient quotes to produce raw data. Based on the classifications by (SS), she then determined whether she was in agreement with their allocation to raw data themes and noted where her allocations differed. She also noted where she identified additional raw data and to which themes she would allocate these. The two analysts discussed this first stage of analysis, and agreed on some changes to the allocations of raw data to raw data themes, and on the addition of new raw data to some themes. The analysts then discussed the initial identification of first and second order themes. SS independently reviewed the modifications discussed during peer debriefing and incorporated those he felt were appropriate into the next interpretation of the data. Subsequently JH further challenged the categories identified, leading to the final thematic interpretation produced by SS. Member checking was also used, although only to a limited extent as only two participants engaged with this process. This involved forwarding interview transcripts to participants for review prior to data analysis. Those who responded verified the transcript content and none replied asking to withdraw their comments or objecting to their interview content.

**References**

1. Rubin HJ, Rubin IS. Qualitative interviewing: The art of hearing data. London: Sage Publications 2011.

2. Braun V, Clarke V. Using thematic analysis in psychology. Qualitative Research in Psychology. 2006;3(2):77-101.

3. Guest G, MacQueen KM, Namey EE. Validity and reliability (credibility and dependability) in qualitative research and data analysis. Applied thematic analysis. London: Sage Publications. 2012:79-106.

4. Miles MB, Huberman AM. Qualitative data analysis: An expanded sourcebook. London: Sage Publications. 1994.
