# Supplementary material for: Patients’ experiences of coping with Idiopathic Pulmonary Fibrosis and their recommendations for its clinical management
Source: PLoS One. 2018 May 23;13(5):e0197660. doi: 10.1371/journal.pone.0197660 (PMC5965862; doi:10.1371/journal.pone.0197660)
Supplement: S2 File — Interpretive frameworks. (DOCX) [file pone.0197660.s002.docx]

**Discussion. Interpretive frameworks**

As the psychological implications of an IPF diagnosis is an under-explored area, we analysed our data inductively to allow themes to be generated from the data rather than using a deductive theory-driven approach [1]. Based on our emergent higher-order themes it is apparent that two key theories present particularly useful frameworks with which to interpret our results. These theories are 1) Ryan and Deci’s self-determination theory [2,3] and 2) Folkman and Lazarus’s transactional theory of stress and coping [4,5]. In brief, self-determination theory (SDT) is a theory of human motivation and personality in the social context; it is further explained by six mini-theories, one of which considers the role of basic psychological needs in determining our well-being and positive functioning. These include the need to feel competent, related, and autonomous [2]. When need-satisfaction is thwarted our well-being and functioning are adversely affected [2]. Competence refers to the need of an individual to be effective and capable in their social environment; Relatedness describes the need of an individual to relate to, interact with, and experience the care of, others; and autonomy refers to the need to be independent and able to control oneself and one’s life. These three psychological needs are universal and their fulfilment leads to psychological well-being, which is reflected in physical well-being and vice versa [2,4]. The usefulness of these theories to explore chronic health conditions has been demonstrated previously. For instance, Rahman et al. [6] used SDT to analyse motivation and need satisfaction as predictors of psychological and behavioural outcomes in patients presenting with a range of health conditions who engaged in an exercise referral scheme. A further study by these authors involving cardiac rehabilitation patients reinforced the value of SDT for understanding the experiences of patients who are suffering from chronic conditions [7].

A brief explanation of the transactional model of stress and coping forwarded by Folkman and Lazarus [4] will illustrate its complementarity with SDT, thereby supporting our use here of both theories to improve our insight into patients’ personal experiences of IPF. These authors proposed that stress is not simply an emotion but also the outcome of an ongoing relationship between the individual and their environment. More specifically, when an individual encounters a stressor (such as an IPF diagnosis) they will evaluate the impact of the stressor in relation to personal objectives in their life. This process is termed appraisal, of which there are four possible evaluations: Harm/Loss (referring to damage already caused, e.g. being diagnosed with a terminal disease), Threat (referring to potential harm, e.g. an upcoming medical test), Challenge (the event offers opportunity for personal growth/achievement) and Benign (the event is not stressful and no action is taken) [4]. These appraisals are not mutually independent and could occur simultaneously at any given time when faced with a stressor. Lazarus states that coping is a cognitive and behavioural effort employed by a person to tackle demands that are created by the stressors [5]. He further theorises that different stressors will lead to the use of different coping mechanisms (problem-focused and emotion-focused). Problem-focused coping mainly aims to resolve the practical problems associated with the stressor on a daily basis. These include changing lifestyle through seeking information on the stressor, or planning and prioritizing [8]. Emotion-focused coping revolves around managing unpleasant feelings that are caused by a stressor - for example isolation, distancing, managing hostile feelings and wishful thinking [4,8]. Additional coping mechanisms that we looked for in this study were avoidance-coping (part of emotion-focused coping) and self-regulation and proactive-coping [9]. Avoidance coping is defined as an effort to avoid facing the stressor and could in itself lead to depressive symptoms [9]. Self-regulation and proactive-coping involve determination of potential future stressors and taking action in advance to negate the impact of these [10]. The transactional theory has been used across a range of contexts, for example to understand coping strategies following disaster [11] and the stress and burnout experienced by French elementary school teachers [12].

**References**

1. Thomas DR. A General Inductive Approach for Analyzing Qualitative Evaluation Data. 2016.

http://dxdoiorg/101177/1098214005283748.

2. Ryan RM, Deci EL. Self-determination theory and the facilitation of intrinsic motivation, social development, and well-being. American Psychologist. 2000;55(1):68.

3. Selfdeterminationtheory.org - An Approach to human motivation & personality [Internet]. 2017. Available from: http://selfdeterminationtheory.org/.

4. Folkman S, Lazarus RS. Coping and emotion. Psychological and Biological Approaches to Emotion. 1990:313-32.

5. Lazarus RS. Emotion and adaptation. Oxford University Press on Demand; 1991.

6. Rahman RJ, Thogersen-Ntoumani C, Thatcher J, Doust J. Changes in need satisfaction and motivation orientation as predictors of psychological and behavioural outcomes in exercise referral. Psychol Health. 2011;26(11):1521-39.

7. Rahman RJ, Hudson J, Thogersen-Ntoumani C, Doust JH. Motivational processes and well-being in cardiac rehabilitation: a self-determination theory perspective. Psychol Health Med. 2015;20(5):518-29.

8. Ntoumanis N, Edmunds J, Duda JL. Understanding the coping process from a self‐determination theory perspective. British Journal of Health Psychology. 2009;14(2):249-60.

9. Holahan CJ, Moos RH, Holahan CK, Brennan PL, Schutte KK. Stress Generation, Avoidance Coping, and Depressive Symptoms: A 10-Year Model. J Consult Clin Psychol. 2005;73(4):658-66.

10. Aspinwall LG, Taylor SE. A stitch in time: self-regulation and proactive coping. Psychological Bulletin. 1997;121(3):417.

11. Matthieu MM, Ivanoff A. Using stress, appraisal, and coping theories in clinical practice: Assessments of coping strategies after disasters. Brief Treatment and Crisis Intervention. 2006;6(4):337.

12. Laugaa D, Rascle N, Bruchon-Schweitzer M. Stress and burnout among French elementary school teachers: A transactional approach. Revue Européenne de Psychologie Appliquée/European Review of Applied Psychology. 2008;58(4):241-51.
