# Supplementary material for: Patients’ experiences of coping with Idiopathic Pulmonary Fibrosis and their recommendations for its clinical management
Source: PLoS One. 2018 May 23;13(5):e0197660. doi: 10.1371/journal.pone.0197660 (PMC5965862; doi:10.1371/journal.pone.0197660)
Supplement: S3 File — (PDF) [file pone.0197660.s003.pdf]

.

PATIENT INTERVIEW TRANSCRIPTION

(patient 1)

Age: 81

Conducted by: JH, SS

START OF THE TRANSCRIPT

Interviewer: What do you think caused your IPF condition?

Patient: In my younger days there when I started working up the promotion ladder I was a junior charge hand, it was a working position, a working charge hand and one job that I had to do; (it all sound double dutch to you) there was a small ladies section 10, but there was a small course that was made of sand, they are going to overhead they baked to 600 degrees, 34 dive down, if they want to go around twice they twice and they come off. I may have to be sprayed with a mixture of water and coal dust. So for the time I was being work for the 1st two hours, even though you are given a mask you spit out after two hours it was solid black. But I think I get paid for the job and I did it for the best part of two years, and I think the amount of cold dust I must have swallowed then. That's one thing.

But the last job I had was in BP chemicals. Obviously we deliver petrochemicals and in one occasion I had a very bad report, where we used to work with Benzene, and at the end of each late shift at the end of the month we would be tested for our urine. I had three tests taken and had results over a hundred and they should be around 100 or less, so they said if that continued they would have to kick me off that job but it felt away and I was alright.

But when I spoke to the doctor down there, I asked what target you really want, was it bellow 100 and he said really we would like zero he said. So they really didn't want that in your system at all. So I think one of those two

Interviewer: So it sounds like, well I guess you got a history in you know in those kinds of environments that might cause, so were you surprised that you were diagnosed with IPF

Patient**:** Yes, but they weren't sure at 1st, I suppose they suspected lung cancer or either it's a form of lung cancer, they had no idea and I didn't particularly want to know. I've got a friend with lung cancer at the moment. By coincidence, about two minutes before I was diagnosed there was an article on the paper about this young man who was about 45 who said he now can't play with his children and things because he got this, and I read his diagnosis and I thought I felt exactly like that you know and I've never heard about IPF and when they diagnosed me I thought I've got what this chap got and he is only 45 and I was already what? 76. And umm I've had a good run anyway, I mean because my heart, the time I found about my heart was when I was in national service as a young man, because as an apprentice you don't go at 18 and the at the time you finish your apprenticeship you were nearly 21 and I was engaged to my fiancé and she was going to wait for me till I do my two years of national service etc. etc.

I was fit as a fiddler, I was playing rugby, soccer and I used to do running 400, 800 meter, I was a fit young man, or so I thought. And I went down and I was grateful. So they told me that I have a murmur, a leaking valve, and meant nothing to me at the time till I was referred to [] hospital, this goes back to 1956. So it was a long time ago and I was diagnosed with a leaking valve of course they didn't do heart surgery in those days, they couldn't stop the heart and um and I had to give a chance to my fiancé to drop me as I was not a very good prospect then, she didn't I'm glad. Anyway at that time I didn't know I was going to life till 40, 50, 55, 60 if I was very lucky but at 81 I am still talking to you.

.

Interviewer: Ya, really you are still going strong.

Patient: I lost my wife 15 years ago, she had leukaemia. If she would have been there she would have been a very attractive girl and pretty talented, sang semi-professionally for 35 years. She was a non-smoker as I. when I say I'm a non-smoker it not that I 100% smoke, for about perhaps 10 years I know I dint like cigarettes as such but when I have a couple of pints particularly when my wife was singing with her band and I was with other people and ide have couple of pints and my wife had to drive me home, anyways I was enjoying a cigarette or perhaps three but I wasn't an addict, I wasn't addicted I was never ever addicted, I just had a couple of cigarettes. Perhaps I shouldn't have because even that could have caused it.

Interviewer: Well you don't know do you, there are so many factors like you say all the industries that you have worked in

Patient: My wife told me one day when I was smoking, you know you look like a monkey with one of those things, and I stopped and I smoke never again for 25 years.

Interviewer: if you were addicted you could have never stopped like that.

Patient: I'm talking too much

Interviewer: No no that's ok, that's what we are here for. That's lovely, we are really interested to hear. So when you were diagnosed it was still a surprise.

Patient: Very much a surprise. Particularly by confusedness in to what it was exactly. Of course I soon learned and told them don't hold back, I don't want to hold back, I want to know what it is as it is? And they said they don't know. And I said what about this article, I read that article and later on I saw another article found on internet and I bothered my children to do it and I've read enough to realize that on average there is a three year life span and some are lucky and get a bit further and others have 8 months. So I knew it was a wide scope that I've got. Anyway I'm glad to say I've got lot of friends and when I was diagnosed I was struggling as I have never been struggling with walking and it coincides with onset of this one. And I'm thinking it may be this which is making my walking difficult. And I told I've been given a bad diagnosis and I have a limited time and some say you are bloody around u said you are going, I'm glad that I'm not but sometimes days I don't mind going.

Interviewer: So you said a few things which were quite interesting there, so when you 1st got your diagnosis and you found that it was a varied life span, how did you feel when you heard the news about your condition?

Patient: I was determined to live my life for the fullest, for as long as possible. By this stage, I said I've lost my wife 15 years ago, after a couple of years I was terrible and I lost my way, I had to be treated for depression and I started out I was missing the ladies company, I'm not about the physical side, I had a couple of relationships one was almost five and a half years. I was quite surprised by the diagnosis and that there was no treatment at all. I thought surely there is something. I'm on painkillers now but those are for my back not my lungs, they may be allied, I have no idea. It is surprising that there is no treatment as such and now I am more aware about the situation particularly after joining this group (support group). It is nice actually to meet some people with the same disease, I thought at

.

least I was not alone. But when I was up there last Monday I couldn't help thinking that this is like shipman's waiting room, waiting for each one to pop off you know. Because there is always news that somebody is gone and that's a person u know and I think I my name would be there one day wouldn't it.

Interviewer: Ya I mean the fact that there is no treatment for it, did you kind of deal with that in a certain way? Have you tried to approve things?

Patient: I understood the few treatments that they did have in mind like lung transplant and etc. it was completely out for me at my age anyway. And with the new laws to transplants from people, I'm for it personally, because if you can save somebody else's life you should.

Interviewer: So I mean you mentioned previously you are a sportsman and you are very active and so on. And you mentioned you use your walker now, how does the condition has effected your daily activities?

Patient: Oh greatly in fact. My dog died two and a half years ago but one of the delights of life was walking my dog. I think he went at the right time because I'm not able to now. I couldn't look after him, I couldn't brush him so in a way I'm glad that he went. But that aspect of my life, just going out for a walk no problem up until six months ago. I still drive and once I'm settle down I'm ok and once I get to somewhere I have to plan everything. Even looking after myself here. I don't eat a lot at home because I eat with these ladies but I would be doing lunch my self today. And I my dishes and I try to avoid walking as much as possible. You got to be thinking all the time you know and that thus affect me. And another thing, because of my interest in sport my wife took me bowling and I was bowling up till two years ago three years ago and I joined the club eventually for about five years and I miss that the companionship. My son-in-law is the captain in the team and I would have loved to play under him. But I'm still, I've got a season ticket to the swans and I'm going there by my wheel chair because I've graduated from an excellent seat up in the stand to lower regions until they found me a spot on the side, and I've been lucky, I've got a good spot there and I've been watching them for the last two seasons. I've got a ticket for the next season but weather I'm there to see it or not, I don't know. One of the things I've found particularly after losing my wife I've never got board because I've always got things on whether its singeing, sports or general life I've never been board, I think the boredom will kill you. That's why I don't like staying in too much. I honestly feel that it has helped me because I would rather go with a band than sitting here waiting for it.

Interviewer: you've been talking about staying active and looking to the future and planning things for the future. I guess it digs in with couple of these questions, on a day to day basis how does having IPF make you feel.

Patient: I attend chapel every Sunday morning, I'm not the best Christian. I've had heart issues since I was 30 and even for this I don't feel paranoid, because I keep thinking about other things. I've started staying in a bit more. But I get up night to go to the toilet and I get pain I don't think its IPF but I might be wrong. I went to a course of physiotherapy and honestly I think it's too much for me, when I came home I was knocked out I wasn't even able to do simplest things. Now my strength is gone and sometimes I feel ashamed about the things I can't do, perhaps it could be because of getting old.

Interviewer: I want to come back to the support group. But you've mentioned the rehabilitation programme, so when that was proposed to you, your 1st thoughts were that's too much I can't do that?

.

Patient: No actually, I thought my input will be valuable because the people there are surprised that I've lasted five years. And I thought I can't actually take part in physiotherapy, I'm pretty sure I can't also because of my heart problem.

Interviewer: you mentioned the support group and you mentioned some positives and negatives being involved in that

Patient: The positive is that I've met people slimier, some of them are very nice. There was this one lady who I felt I really cheered her up. I would imagine she is approximately my age and I said to her I've been diagnosed for five years. And she said h Fred had it for three years (her husband) and I felt that she's thinking that there is hope for Fred for another couple of years. So that's the upturn. Downturn is every week they announce the deaths to me. And that is a bit of a down turn because u all know that you are going sooner than you like to be. Another down side I felt last time I was there. I went there fairly early and as people were coming in a chap came and sat with his wife and it was his first time and he wasn't getting involved and I thought I felt like that in my 1st time and I didn't feel that good coming out of that one because I thought do I really have to come here and perhaps get depressed. But I kept on going because if we could do anything for 20-30 years down the line…who knows.

Interviewer: you said a couple of things that I just wanted to follow up on, you said about you don't like to live with it but you have to live with it, I'm just interested to know how do you live with it? Is that a question you could answer?

Patient: Putting it out I don't mind, oh no that's wrong, I do mind it every day. Challenge is living with it. As I described earlier, finding the simplest way to do things and I try to keep everything in precise order. So things at the same place all the time. Matter infect the lady who come on Friday said you are so obsessed with everything but I find life is easier that way. That is also because of my mind and I'm a great believer in keeping the mind occupied, I mean I'm not a couch potato when it comes to telly but I love watching interesting programmes.

Interviewer: going back to the support group, the thoughts that time when you went and thought you didn't want to come again. Was that because it reminded you how you feel, you know about your condition?

Patient: May be, it's just the days because the feelings were too varied. I got days where I'm feeling reasonable and life is full of hope and there have been days where I thought I won't see tomorrow when there is no company around me, those were the down days. I didn't have lot of those but I don't get deeply depressed though, it's just that accept the fact that my time is limited but sometimes going to support groups brings some hope to you, you know because everyone else's is time is limited. So yes I'm glad they got it and I certainly will support it, I might miss out occasionally but I will go as long as I'm able to and it surprising that new members are showing up all the time. So it's a good set up and yes it been ignored probably because many medical professions doesn't understand it. They don't know what is causing IPF, they are guessing its work, smoking, genes, life style, I've always enjoyed a pint but I'm not a boozer, I've never been a smoker as such and I've always been reasonably fit.

Interviewer: You talked a little bit about lack of awareness in medical profession about IPF, what causes it you know and so on. How does that make you feel, the fact that there is so little understanding and knowledge of your condition?

Patient: The patient did not give an answer relevant to the question

.

Interviewer: You know, you sort of mentioned that it's an untreatable condition has anybody suggested any ways to help you manage it to make life more comfortable and prevent the decline?

Patient: In a way yes. Because I've learnt in two occasions in the last five years, I've had people come and install things like grab handles in my shower and alternate steps from a larger step to a smaller step, those type of things. Things like that help you with life. This thing was given to me (walker). But do I like too much of items, No, but I'm old now and I can't get around here without any support. And I've got two alternate sticks if it is in and out of shops or the club. I got a walker and a wheelie and I've got an electric one out there, second hand I've bought it and I would love to go around doing walks with my dog,

Interviewer: do you rely on other people?

Patient: I've got a gardener, he was meant to come last Thursday but he hasn't. I keep the back gate open but I don't like doing, in case he turns up. And then I've got two daughters and a son, they are always there if I call them but one of my daughters is in Scotland but she would be down in the next plane if necessary. So I'm not short of support. I'm very sorry for people who haven't got back up and I've got a couple of those.

Interviewer: you mentioned your family, how did they supported in your condition. Did they encourage you to do anything to look after yourself?

Patient: They've always said to me, I would never have to worry about being on my own. But I want to be on my own as long as possible. Even as you get older you like to have your independence. I like to feel the king of my kingdom. They would do anything if I ask my family but they prefer me to ask them because they know what I'm like, I'm stubborn. I couldn't have asked for a better daughter in law for my son, unfortunately she can't have children, there in their 40s now and she is excellent. She always cared about me. And I've got these systems, my daughter who is a teacher sends me a text "OK Dad", and she knows that I'm OK. So there caring and we got these system because there is nothing worse if you collapse and you are on your own. So the family is so good being not pushy.

Interviewer: I just want to ask you a few questions about pulmonary rehabilitation, so that was something that you felt, you don't have the strength to do?

Patient: I don't think so

Interviewer: what's your view on what it includes?

Patient: Going by what I did with the heart, I guess they are using some weights, running or semi running and the rowing machine, bicycle and all, I enjoyed doing all that, and it did me good and I know that it will probably do good to me now.

Interviewer: I think, the rehab programme, if you were offered that a while ago, right at the start of your condition, do you think you would have felt differently?

.

Patient: Having a look back most patients haven't got my back problem.ve got these elastic bands at the back for exercise, I don't do it to be honest because I'm already overtired to what I am. So I think it's a case of yes it would do me good or it would or it will finish me off. But if it was for other physical things I would be the 1st to say yes

Interviewer: when you were talking about different types of exercise that you did in your cardiac rehab programme and then you talked about doing seated exercise with your therapy band, if you were to design a rehab for programme for people with your condition, what would you recommend that it would include?

Patient: Some patients are 40- 50 and most of them are fitter than me. I think it's a good thing and I think people should be encouraged to do it. And if it was 10 -15 year ago I would have given it a go.

Interviewer: are there any other thing that you think will be useful to include? I mean you mentioned seated exercise, is that something that would be helpful?

Patient: There are quite a lot of exercises that I know that you can do sitting down. People might be put off coming or might be encouraged by sit down for these things, there are plenty of exercises that could be done sitting down.

Interviewer: I suppose this is a bazar question for me to ask, but obviously it's an untreatable condition and the exercise won't cure it so from your point of view, what would be the benefit of exercise with somebody with IPF?

Patient: I think it will prolong your life, because the fitter you are the more it's going to help your lungs anyway. There are lot of people who don't realize that. The other side of the coin I've seen people dropping dead during their walking, and they are quite fit people. So it doesn't automatically mean if your body is fit you are not going to pop something else. But basically I do subscribe to keep as fit as possible. I've done it by not necessarily doing exercise but being active (doing daily activities like cutting grass). For those who would say I want to sit down, certainly there is enough can be done.

Interviewer: and part of the rehab programme is kind of education about IPF, you've had IPF for 5 years, which is a long time, what kind of things do you think will be useful to educate people?

Patient: I'll show you, because I write to different charities (shows an article on IPF by BTS). You know that was sent to me, it explains what it is and implications of it. I think something like that could be handed to people so they would understand it, particularly in the early stages.

Interviewer: so do you think information is useful, and you've got a case study here on somebody called John, he's got IPF. Was that helpful for you to have read?

Patient: In the early days when I knew nothing about it, I thought it was only matter of years with it that would have been very helpful, as some people are. Lot of people need to know what the disease is and understand it and they can accept it then. You understand the news and you know you won't be around in 7-8 years' time. Unless it's somebody who disintegrate with the news, as long as people know the full facts, you could expect it, life with it and live accordingly. I lived normal as I could.

.

Interviewer: what would be your advice to somebody if they were diagnosed today with IPF?

Patient: You should find out more facts, face to the facts, accept what is inevitable and live accordingly. Whatever you want to be, if you want be cocooned in the house watching telly so be it. I don't subscribe to that but try to keep your mind active as well, as I always believe an active mind helps the body.

Interviewer: Do you think having IPF have changed you as a person?

Patient: It's a damn darn question. I like to think, I've got lot of friends who show lot of affection towards me, and certainly I'm bit more short tempered now, partly because I feel bit of a grievance that why this after all the years of struggling with my heart and everything. I think I'm the same person I used to be. Lot of people like to see me especially the ladies.

Interviewer: as an IPF patient was it easy for you to get hold of all the information? How did you find that process?

Patient: 1st of all there was that paper which gave me insight before I was diagnosed, I've never actually been given written information other that what I have picked up myself. Information is good and eventually more people would understand what this is. Lack of information always causes more worries thank being told the hard facts. No one has given me enough physical advice on it. Not everybody want to be known but I do.

Interviewer: Thank you for taking part in the study

END OF TRANSCRIPT

PATIENT INTERVIEW TRANSCRIPTION

(patient 2)

Age: 80

Conducted by: JH, SS

START OF THE TRANSCRIPT

Interviewer: can you tell me a little bit about the onset of your condition and a little bit about it?

Patient: looking back I think probably about three years ago, we were on holiday and then um, and I happen got a chest, never thought more of it. And then we went on a cruise, 2014 February/march and again I had a chest problem. And we went to Spain, I had a really bad chest there and I was putting it down to picking something up from travelling on the plane. I went to see a chemist and she prescribed antibiotics, which took and when I got home I went to see the doctor and he was a bit off and asked why I hadn't gone to see a doctor in Spain. And I said well I've seen the chemist which was free of charge and she did the job well, she gave me the anti-biotics which worked but I came to see you as my own doctor. So anyway he checked me over and he wasn't sort of very keen on going any further. My wife was with me at the time and she said this has happened two or three times, so in the end he decided that I should go and have a chest x-ray. Following the chest x-ray at the singleton hospital and the consultant referred me to Dr. [] in [] Hospital. When I saw he came out and said I have pulmonary fibrosis. And since then I've been seeing him fairly regularly.

Interviewer: and then did you get much information about pulmonary fibrosis?

Patient: I firstly understand that there is no cure, it's a disease which lot of people don't recognise and my doctor was a little short at the time but after he found out that I was diagnosed with this he was exceptional after then, especially at the time my wife had a brain tumour and his attitude from then on was absolutely fantastic.

Interviewer: but initially is was like a mystery it sounds like?

Patient: It was a mystery, he didn't think somehow, though I was complaining about chest, I know one thing I've missed out a lot now just thinking back, a year last September (2014 September) we've gone to Madeira and we were swimming normally, and we are both good swimmers and I would swim behind my wife and being a man I would always have to accelerate and pass her, but I found out that I was struggling to do a length. That's I've supposed why. I went to see the doctor and said there is something wrong because I've never had any trouble swimming at all or distances, that was the main thing you know

Interviewer: so you were quite active before?

Patient: Oh yeah, I used to until this, I'm on crutches now for the last three years and I've had balance problems before and I used to play a lot of golf, swimming, cricket you name it. Ive had no problem, but now I'm a television viewer.

Interviewer: so there has been a change in your activities levels

Patient: I find I'm tired if I got down to put my shoes on I become breathless. The other problem is my balance and walking and I find it a great struggle in the last two years I've gone from a wheel chair which I use, because when we are away with my friends I was slowing them down, so I got a wheel chair and my wife had a problem pushing it because she had arthritis in her hand, so I got a scooter.

Interviewer: other than the way you get around in places and getting breathless when you bend down to do your shoes, how else has it affected your daily activities?

Patient: I would say, people would say I am lazy but when I walk to the kitchen now I leave plates, knives and everything, where I'm preparing the meal, I'll leave them and wash them all in one go and put them back, rather than put them away because I'm not going to get up and walk 5 yards to get the plates and come back. I've sort of try and do everything in one place so I don't go somewhere and I would have to back for something, can't be helped but sometimes you forget, I sort of, it's like I go in to the lounge and the kitchen, I always go in the bathroom because I think of getting to the lounge and I don't wana go back because it not on the way, it's sort of reorganize your life, try and do everything in one place before you move to another.

Interviewer: is there any other way how it affects what you do and where you go?

Patient: The next doors neighbours invited me few weeks ago on a Saturday night, because I was on my own at the time and I was coughing quite a bit which was a bit embarrassing and it seemed to persist and I thought this is no good but um I couldn't do anything about it. That's the problem, I find quite often at night I start coughing and sort of like there is medicine. The only thing that I found helpful is menthol sweets which seemed to help a little.

Interviewer: Is it only you in Swansea or do you have your family here

Patient: no I'm on my own here. (Rest of the answer is deleted from the transcript because it was off topic and irrelevant)

Interviewer: What does having IPF mean to you? As an individual.

Patient: um, I suppose really it's just something carped on over the years. I've always like doing everything by myself. I've always been very sporty, now I don't go golfing, I don't play tennis, I don't go swimming, it's limited that side, the only thing is I can walk in water because I've got crutches so I'm OK in the swimming pool, but I suppose now I need somebody with me and I'm general it's sort of, wouldn't say its lazy but I tend to leave things for a bit till they all get together and as I'm on my own now I just please myself. It's like I don't make the bed every day because I can't stand so I leave

it and I would leave my clothes on the side of the bed at one end so they are ready for me to put on in the morning (Rest of the answer is deleted from the transcript because it was off topic and irrelevant). I suppose everything is very slow but one has to accept that's the problem and make the most of it.

Interviewer: How do you make the most of it?

Patient: I suppose, since I came back from Canada, there has been a lot of sports on so I've tend to watch that and left things which is needed to be doing, these days there is not much in the television now so I'm getting on with my paper work. Last few days I feel a lot better about myself. I've seen Dr. [] three months ago the day my wife passed away. (Rest of the answer is deleted from the transcript because it was off topic and irrelevant).

Interviewer: Is there anything positive come out from the diagnosis of IPF?

Patient: Difficult to say isn't it. I've lost my wife and I'm still alive. So that's positive. (Rest of the answer is deleted from the transcript because it was off topic and irrelevant). When people see you coughing they say take cough medicine, take this take that, they don't understand it.

Interviewer: Does it make is sometimes difficult then, because people doesn't understand it?

Patient: Because it's invisible, they think, or get some cough medicine or do this and that, when I called Dr. [] it was completely different. I don't think they believe there is anything wrong with you in a way but quite often they would say why you are panting when you are doing nothing literally, or you don't realize you are.

Interviewer: How does that make you feel?

Patient: When my wife was in the care home and I used to sleep and I used to have a phase about two weeks when I felt out at night, as soon as laid down to go to sleep, I was getting sort of, I couldn't breath properly or had that feeling that I wasn't able to get my breath which I found difficult to handle for a bit. That's disappeared now. I didn't know if oxygen was working at the time, but it is worrying when you are on your own. Every time you lie your head down and in 10 minutes you can't breathe properly and it's a bit worrying and you wonder if it will get worse.

Interviewer: What is your understanding in pulmonary rehabilitation is?

Patient: I've had a leaflet from lung association and about exercise and I have not done anything about it now. There is one in Swansea. I do go to a physio class, I go in twice a week now doing regular exercises and I can agree with that. I can see doing all the exercises without being a couch potato and it's good.

Interviewer: so what kind of exercises can you do, could you give me couple of example?

Patient: yes you do marching on the spot moving your legs to each side forwards backwards, starches and walk one foot in front of the other which I can do with support. Some of them I can't do like I can't get on my toes or heals but that due to my balance problem. They work through your whole body like exercising your arms use the tuber to put pressure to your joints, especially to your arms and wrists. You do exercises where you do stretch out the tension and hold for five seconds and repeat it for 5 times. I can understand it and what it doing.

Interviewer: When you said it was doing you good, what type of benefits do you think you got?

Patient: I can't walk very much, everybody says you should walk here walk there so it has given me exercises to do with 10 or 12 people and you tend to say right, I'll do it all I'm not going to sit down you know not give in, otherwise in sitting in all the time. I can't go out for a walk, I'm struggling with my crutches and the idea is that it will tired you for a bit and get the heart rate going and get your lungs working and everything.

Interviewer: Do you think it's kind of helping with your condition?

Patient: It could be because, I don't know how the condition goes and the leaflet you have given me is very nice, it says 50% only make it for three years, mine is staying static at the moment. It's nice to get a reading which confirms you are not deteriorating. And looking back my wife and I took a full MOT few years ago and I didn't take any notice of it but they said there is spot on your lung which could have been the beginning of it, as I thought I don't smoke and I was fine for years afterwards but it come on in the last 18 months, it must have been there and the last few months I have been at a point where nothing is happening. But I'm reasonably happy ATM.

Interviewer: do you actually enjoy the exercise sessions that you do?

Patient: yes, the only thing I find, the original one I went to was run by NHS for 8 weeks and at the end of that you pay as you go, I think a couple of pounds. The 1st one had tea and biscuits and at the end of it you get talking with other people, especially if you are sort of a single person in your own it's nice to have some company. But the second one there are no company you are on your own. It gives you something to look forward to otherwise you will be sitting in home all day.

Interviewer: If there was [an exercise class] at [] would you be interested to attend?

Patient: yes

Interviewer: what kind of thing would you like in it in terms of exercise?

Patient: well, I'm in the dark about what you can do to help the condition and I'm open to suggestions you know, anything that might help is going be good.

Interviewer: What would be the things that would put you off going like exercises as you said?

Patient: Nope

Interviewer: are there any kind of other exercises that you like to do or environments may be like mixed groups?

Patient: yes it doesn't worry me I mean in the past ive done exercises and ive been the only man around 10 women.

Interviewer: You are open to exercise so how often do you think you can manage doing exercise on a weekly basis?

Patient: I've only started to going for the physio twice a week instead of once. But to me pulmonary one would be more important compared to the physio one so I would choose pulmonary over physio. Rest of the answer is deleted from the transcript because it was off topic and irrelevant)

Interviewer: Do you think there would be wider benefits doing pulmonary rehabilitation?

Patient: It's meant to make your breathing easier whereas the other one is a more arthritic one on joins and muscle.

Interviewer: You mentioned your family in Canada, do they have any view on about your activity level?

Patient: no they have never said anything at all. You can't expect me to do a lot, if I need them they are at the end of the phone. But they have their own life.

Interviewer: If you can see yourself going on the pulmonary rehab programme, are there certain things you look forward to or something might worry you?

Patient: I don't think that anything will worry me. I've always thought myself as somebody who doesn't worry and things happen in life and you get on with it.

Interviewer: because you wasn't aware of the pulmonary rehab programme, you haven't talked about it with anyone?

Patient: No. the only thing I got was a leaflet from British lung foundation saying about classes but that's it

Interviewer: when we talk about pulmonary rehabilitation there are different components like exercise and education. What kind of things would you like to see in it or is exercise is just enough to you?

Patient: to me exercise seems to be the only thing. Unless it explaining the problem what's causing IPF there is no point. I'd rather be told things you know.

Interviewer: If you were given a choice of dong an exercise sitting down, would you…?

Patient: I find it easier doing exercises sitting down.

Interviewer: so sitting down exercises would encourage you to attend?

Patient: yes probably.

Interviewer: looking back in life do you think may be something that you did caused your condition?

Patient: Dr. [] sort of dug in to this. I had a family motor business and I thought looking back in the sixties when you look at the breaks, when you take the brake drums off and you have asbestos and you blow it off and may be when u paint cars u cam puff it. He said it doesn't causes IPF and this came 40 years after I breathe asbestos (the spot on the lung).

END OF THE TRANSCRIPT

PATIENT INTERVIEW TRANSCRIPTION

(patient 3)

Age: 81

Conducted by: JH, SS

START OF THE TRANSCRIPT

Interviewer: You're IPF, when did it 1st got diagnosed?

Patient: well, I was diagnosed year last May. Year last January / February, I was with my doctor. Think all this after losing my wife in July. I didn't let the grass grow on my feet because I've never been too morbid. In October I went to north of Organya but this year it's so bad I can't go anywhere. But as I said I was going to the doctor in February last year and I said I want to see a specialist and they refused so I booked to see a consultant and on one morning I got a call saying we had a cancellation and a spot opened up so I went to see this doctor in []. He checked me up and said I have clamping fingers, got x-rays and said I think you have IPF. I have never heard it before so he arranged a CT scan and then saw Dr. [] about a year ago.

Interviewer: it has affected how mobile you are, has it affected your other daily activities, way you do things around the house?

Patient: Oh I can't do anything. (The rest of the conversation was deleted from the transcript because it was irrelevant to the question).

Interviewer: driving long distances with your oxygen on is quite difficult for you?

Patient: never tried it. But if I was driving on a cushiony seat and I'm only driving and not talking. This is what rehabilitation is going. If your muscles are fitter you need less oxygen, this is what they do there strengthening muscles and it marvellous.

Interviewer: so you have been doing the rehab exercises have you and for how long?

Patient: I've done that. About six weeks of it but I lost 4 days, 2 weeks.

Interviewer: how did you find it?

Patient: well, as I said to you earlier what they teach you are the basic exercises to keep your muscles toned up. By toning your muscles up you get stronger muscles and use less oxygen up. It's easier said than done sometimes, if you are struggling for it. I mean a year ago I was doing all the grounds on my tractor cutting grass but this year I can't do anything.

Interviewer: what kind of exercises did you do in the class, what could you manage?

Patient: we would sit in a chair, they put weights around your legs and lift it up like that and you stand up and sit down and you step up and stretching and bicycling. I did what I could.

Interviewer: did you enjoy it?

Patient: yes because, I found it struggling but as they said to you do it to your own phase. They never pushed you. Yes and it was about meeting other too.

Interviewer: some people actually do not go to rehab programmes, what encouraged you to go?

Patient: I told myself you have to try anything and everything. I have an oxy meter and I check it during my activities.

Interviewer: when you were doing the exercise session, do you ever worry that your oxygen levels would get too low.

Patient: no because I'm on this and if I feel like it I turn it up a bit and I take a full cylinder when I go.

Interviewer: you said about doing exercises, you know doing what you can. Do you think it helping your condition other than the extra oxygen going in to your muscle?

Patient: I don't think so. I just don't know. I know the difference, from last year it it's a huge difference and I've gone down.

Interviewer: How do you feel having to use oxygen all the time?

Patient: I've got no choice you know. It's like a tie. You are stuck at an end of a tube really. I try and manage without it. I don't want it any more than its necessary you know unfortunately. I don't sleep with it so I go down quietly.

Interviewer: how does your family help you? Do they help you to manage your IPF?

Patient: My daughter came in some time ago and helped me to slip in to bed. (The rest of the conversation was deleted on patient's request).

Interviewer: going back to your family, do they encourage you to be active or do they say dad sit down, take a rest?

Patient: well, it's not a matter of encouraging me because they know what sort of a person I am. If I can do it I'll do it. I'm a very independent person.

Interviewer: what do they think about pulmonary rehabilitation, do they think it's a good thing?

Patient: I don't know, I haven't talked to them about it. They know I'm going and I have finished it now.

Interviewer: having gone through that programme. Do you think its missing anything? Doesn't necessarily have to be exercise, may be lifestyle or diet?

Patient: No, because you see, you did an hour of excising and then you are sitting down having a cup of tea and biscuit and chatting with others. There was a dietitian there and I saw her because I got a stomach problem as well. I don't know if it affecting. I had that for a long time (rest of the conversation was deleted because it was irrelevant).

Interviewer: do you believe that something you did caused your IPF, like smoking?

Patient: never smoked other than a few cigarettes as a young boy. Never was a drinker, drank very little. If you google IPF you would find acid reflexes could cause it and it makes sense to me because I've suffered from it in the 90s. So maybe it's a contributing factor. I don't know

END OF TRANSCRIPT

PATIENT INTERVIEW TRANSCRIPTION

(patient 4)

Age: 71

Conducted by: JH, SS

START OF THE TRANSCRIPT

Interviewer: How were you diagnosed with IPF?

Patient: Here comes the start of the problem, In February, 6th of February I fell down the stairs from top to bottom and went to [] hospital. They were going to operate and they found me not fit enough to undergo anaesthetic and I was in there for a month. Mr [] did a multitude of tests and eventually I was diagnosed with IPF. Gave me something like a short period. It was like months or three years to live. Because it came so quickly and I had no indication that id suffered from this condition. My background is working in an office environment not the same office, but different places for 26 years. I was in for a month, I have oxygen in my house and in my car and after a month I came out, knowing I have possibly a short period to live, I started to arrange the inevitable but I also took to drinking too heavily and end of June I got jaundice. By September I finished taking oxygen and then I started the rehab in September same year, three years ago. It was 7 weeks twice a week (14 sessions). I found it extremely helpful. And that's the introduction.

Interviewer: so you've been through the programme, what kind of experience to you have, the positives, and the negatives?

Patient: I didn't have any preconceived ideas. I had no idea what's it going to be like. They said it going to be a light weight circuit training. It wasn't the important thing I think. It was mentioned that it's not a competition although there were around 10-15 people, you would do exercises to your phase. Yes you were trying to do routines of 10 pullups but if you couldn't to it you do as much as you could. It wasn't to gain strength but it was to exercise your lung and mussels to help your blood and air to go to the lung. There were people who supported it . it must have been very good, well I know it was good. Because the dropout rate was minimal. If you miss a day people were coming with doctor's letters the next day. All thought they should participate. It did get a group of people working to gather with similar conditions. I prefer to say a condition rather than a disease because it sounds less when you say condition.

So no preconceived fears and the results were good. Initially your heart rate and oxygen recovery time were taken and they were periodically recorded just to see if you have improved and most people did, so I was more than happy to participate. But after I completed you couldn't do it again and I guess that's a draw back. But I was referred by my GP to group down in Port Talbot. There were all of mature age and was having COPD. It wasn't a gym but they had dumbbells and weights and it was

professionally run, so we did that twice a week and I've been doing that ever since. I find lifting weights easy but anything to do with breathing like waling, I find it difficult.

Interviewer: you don't exercise regularly now

Patient: yes because I'm out of breath when I do. (Rest of the conservation was deleted from the transcript because it was irrelevant). The exercise programme had 14 work stations, 2 minutes at each station which alternate from chest, breathing, legs or arms and it works. All the participants were elderly and it was great listening to them. It's important as well as the exercise because we were all suffering from the condition COPD or IPF and again people like to come talk and socialize and mentally it helps as physically. You don't feel like you are doing for the exercise but to meet other people. I find that rewarding and everybody in the group find it satisfying as well

Interview: So do you find other mental benefits, so is that kind of social connection element that you were talking about?

Patient: I'm not a words smith, I'm more figures and maths. I love the gardens but I can't go much gardening any more, but mentally I feel good. I do loose concentration when people are talking especially when they are talking about death.

Interviewer: can I ask how old you are? Sorry to be rude.

Patient: I'm 71. We had our 1st granddaughter last October. She has a quite a character now. So exercise from rehab I would highly recommend.

Interviewer: would you go for rehab if different types of exercises were offered? You know you talked about warming up circuit training and cool down. Is there kind of another exercises that you would feel you could manage better?

Patient: I don't know what is available. Seated exercise may be beneficial. I don't want it to be one to one because you take away the group feeling. But I will be going back on Monday with my oxygen and I find it easier to do exercises with my oxygen on.

Interviewer: How would you encourage the other people to take part in pulmonary rehabilitation?

Patient: initially I will direct them to our support group. Then I would ask someone who had experience in the condition and rehabilitation programme to talk about rehab, who can pass on their true feelings not a clinical thing, somebody talking from the heart who had done it.

Advertising- there are lots of leaflets with the doctors regarding lung cancer and diabetes. But there seems to be little knowledge in advertising IPF. May be it because it only effects a small group of people. Notices in hospitals would be good. Not making feel frightened from it, it's not a competition, u r not to achieve a certain level but any exercise is good for you. You should be encouraged not forced by the trainers. You shouldn't be the bust but you should be doing your best

Interviewer: it sounds like you have started the rehab very early on when you were diagnosed, you think being active all that time had helped keep you avail?

Patient: that is true. I don't know.

Interviewer: How does it make you feel to have a condition that there is very little information about?

Patient: privileged! I've got a rare blood good. Feel special. I don't think why me. There are positive things a million things that are good. And this s going to be resolved in years in the future although it's now untreatable. I don't have bad days. I have good days and not so good days.

Interviewer: in terms your family, what's their view on you exercising, are they supportive?

Patient: Oh very much so. Extremely supportive. My wife says there is nothing wrong with me but she support me. I love cooking, doing the prep work but I get tired but my wife supports me because she knows I love cooking. My grand child can sit on me but I can't lift her up, is surprising because I can lift dumbbells.

Interviewer: do you think that you did something that provoked this condition?

Patient: I smoked. One a day and was up to 15 a day and 20 a day in the weekends. Then I gave up. My office environment was a non-smoking place.

Interviewer: Thank you for coming today

Patient: we all know we are going to pop out one day, so be it but life is good as we are living it.

END OF TRANSCRIPT

PATIENT INTERVIEW TRANSCRIPTION

(patient 5)

Age: 65

Conducted by: JH, SS

START OF THE TRANSCRIPT

Interviewer: could you tell me a little bit about how you were diagnosed with IPF?

Patient: it was in 2008, didn't realize I had a problem it was found by chance because I had an operation in my eye. Before the operation the anaesthetist checked me and said you have a rattle in your lungs and you really need to get it seen to. So it was diagnosed then. And I've been attending the clinic ever since every six months. It was an oddball in some ways because the results of the lung function tests hardly changed. The Dr. was expecting to see deterioration and it doesn't appeared to have happened, thankfully. When he see me he says I would expect to see deteriorate with your age. I'm 74 you see. And he said I'm not seeing that. From the beginning its being don't change anything. But I put stability down because of the exercises regime that I'm doing, which I have always done. I don't go to the gym, I don't pump ion but I'm active in other ways. I go for a walk for two hours with the dog and its quite tuff walking, you couldn't walk in shoes, you need boots, and it's that sort of walking. And when I do walking with people who haven't got the condition they get you know. So it's kind of vigorous work.

Interviewer: so you didn't had any symptoms beforehand?

Patient: I was having some Flem. But not severely though. And there was a slight cough. Other than that I didn't know really. I was never really out of breath. Also with the lifestyle I mean, now widowed and just having finished work you can imagine how busy I am from Monday and Friday, with the grandchildren, those two days are blown away. I've been working Tuesday, Wednesday and Thursday and that left me Saturday and Sunday to do pink jobs and blue jobs you know. Where previously I was doing blue jobs. There are also lot of house work and stuff like that. So I was busy. I've got three children and they are all in the 40's and I have five grandchildren. And I'm seeing them quite regularly.

Interviewer: what do you do for your occupation?

Patient: I was working as an engineer (a sales consultant) and I was only working for this company for eight years and they offered me a job when I was 65 and I retired.

Interviewer: before that what did you do?

Patient: for lot of years I was hands on machining and for a number of years I worked with graphite. If I was not near a machine I was near a machining facility and there is a lot of dust there. This is manmade graphite with lot of resins and oil.

Interviewer: did you wear a mask?

Patient: Not really, I mean we did but no one did it really. Because it was carbon at it was meant to pass through your system. Whether that was the cause of the damage, I don't know, maybe not. It would be interesting to know about other employees in the industry. (The rest of the conversation was not included because the patient was talking about graphite).

Interviewer: when you were diagnosed were you given any information about IPF?

Patient: only that the disease won't go away. Its life threating. I've seen life expectancy is between three to five years. But it's not my case thankfully. I remember asking Dr. [] if the results of my lung function test is stable, why would you want to see me every six months? And his words were it's a disease I won't trust. I also understood it could be caused by really any foreign body. (The rest of the conversation was removed for the transcript because it was irrelevant). I smoked up until 1969 and in the late 90's early 2000 both my sons were smoking cigars and I would have an occasional cigar, but other than that I never smoked. I've never had a lung injury but had asthma as a child but it went away. I don't think there is enough information out there to make people sit up and think about it and prevent this.

Interviewer: when you were diagnosed, how did you feel about that?

Patient: I didn't get particularly bothered you know. I thought if that is the way it is I should give myself the opportunity I can, stay fit which I have done. And since my wife died I have lost 10

pounds. I thought it was grief but it never came back. It doesn't bother me, I think one of my fails is I have a flat scotch and a glass of wine, I guess that's my only health fault. I don't take any medication, I've got small aches and pains. Last 18 months I have had 5 operations and that itself could be a trauma. But other than that I'm tip top.

Interviewer: so your life style haven't changed much?

Patient: no

Interviewer: has it affected what you are able to do?

Patient: I haven't tried running so far. Every now and then I would go up the stairs and I would think woof, I would feel that. Back in February was one of the worst times with regard to this. I felt really sorry for myself. I think I had a chest infection without any other symptoms. I was walking the dog and I wanted to go home. It was never like that, I could see myself going down a spiral and it lasted about a week. So I was feeling sorry for myself but other than that it never bothered me.

Interviewer: what does it mean to you to be diagnosed with IPF?

Patient: as long as my results are normal I feel that I'm on top. Sometimes it could be a little depressing like during February thinking is it gona incapacitate me you know, when life ends is this what I'm gona die from, but overall not too bad. I'm aware of it daily basis but it doesn't bother me or restrict me. If was diagnosed with lung cancer it would have knocked me over, but knowing I have a fighting chance as things are, it's not bothering me. I worry about my children worrying so that's why I take care myself and it's a concern.

Interviewer: if you were given a chance to take part in an exercise programme would you be keen to take part in it?

Patient: ya, I'm keen to put in anything I can. There could be something which benefits me personally.

Interviewer: are there any particular exercises that you find beneficial that you are keen on doing?

Patient: no, I've never been a keep fit for nothing sort. I've been playing football and all that. I've always done walking and being active. I've had a garden, a house, kids, dogs, so I've always been busy you know. I don't need to go to the gym.

Interviewer: you talked about the physical benefits of exercise, do you think there are any other benefits of exercise related to you condition?

Patient: I don't know. I think it might be beneficial to the upper body. I can't lift things much these days. I've sometimes found lifting whatever across the garden these days, but before it was easy. So that side of it may.

Interviewer: form you're prospective what do you think encourage people with IPF to do exercise?

Patient: when I see some people in the support group I think, you know you are much younger to me but look older to me and I think if it is the condition or the life style prior to the condition. Maybe they have been diagnosed as the same point as I was and did they thought that's it. And when I had that shortness of breath in February it was quite depressing. So if they are feeling like that there is no going back for them. If they were diagnosed at the same point as I was, much encouragement should be given to exercise and maybe role models is what they need. The exercise wouldn't stop the disease but it might slow it up. (The rest of the conversation was deleted because it was irrelevant to the question). When I went there 1st it was depressing may be it was bad timing with things with my wife and all.

Interviewer: apart from exercise and support group do you think there are any other elements to be added to a rehab programme?

Patient: I don't really know. (The rest of the conversation was deleted due to irrelevance to the topic). The other thing is when you tell people about an exercise programme they say this and that, but if it was a walking programme it might be more attractive to some. The fact that you are taking the to a gym environment it might put them off.

Interviewer: how does your family feel about you going on to do an exercise programme?

Patient: they will be like get on with it. They would say I'm an inspiration to them. I don't think anyone would object it. (The rest of the conversation was not included because it was irrelevant to the question).

Interviewer: that's amazing.

END OF THE TRANSCRIPT

PATIENT INTERVIEW TRANSCRIPTION

(patient 6)

Age: 73

Conducted by: JH, SS

START OF THE TRANSCRIPT

Interviewer: thank you very much for coming today. Could you tell me a little bit about IPF and how it was diagnosed?

Patient: Yes, diagnosed in 2006, purely by chance. I had a cough and had two sets of antibiotics and it didn't get shifted as all. So I went back to the doctor and he said you have a lung problem, you should go for an x-ray and see a chest consultant. Then I met Dr. [] and he said that I have IPF, which I've never heard of. 1st thing which came in to my mind was lung cancer because I was a smoker but after having x-rays and biopsy it was confirmed what it was. And I managed it very successfully until March. Up until then I went to the gym twice a week and I was not on any medication, I'm still not on any medication but tomorrow Dr. [] is going to put me on some tablet.

Interviewer: OK so, you were going to the gym a couple of times a week and what sort of things were you doing there?

Patient: the bike, treadmill and occasionally using weights.

Interviewer: when did you stopped smoking

Patient: 2005

Interviewer: so the exercise was helping you to manage your condition

Patient: yes definitely, I stopped going when I was ill and I haven't been back since. I took part in the pulmonary rehabilitation programme in Moriston for six weeks and from there I got referred to another local council.

Interviewer: so you stopped doing exercises on your own

Patient: I used to go for walks but I haven't got energy to do it anymore

Interviewer: how far would you walk typically?

Patient: I walk down the marina a couple of times to get coffee. I walk the dog twice a day and I had no problem. But since March its really knock me.

Interviewer: has it affected any other aspects of your life?

Patient: I've lost some weight and I can't seem to put it back on, that seems a problem.

Interviewer: when you were doing exercise what kind of benefits was it giving you?

Patient: it was keeping my lungs clear but now I have lot of mucus and flem. And it felt better. When I was done you felt that you have achieved something.

Interviewer: so when you said it made you feel better, did it make you feel better about having IPF?

Patient: IPF is a disease I can't do anything about. All I can do it is to prolong it as much as I can. And whatever I can do to do that I will. I know a few people that are going to the rehabilitation course and they complain about being depressed. Well I'm not depressed, vie accepted what I have got. And try to be positive.

Interviewer: Did you feel like that from right at the start?

Patient: oh yes. I mean I possibly didn't realise how serious it was but I got lot of knowledge over the years.

Interviewer: so when you were 1st diagnosed, what kind of information did you get?

Patient: I got information from Dr. [] and the rest from the internet. Since we started the group I realize so many people in the area had the same problem. We talk about how it affect different people in

different ways and for me I find it difficult to walk when it is windy and when it’s hot, wet and cold it affects you. It seems whatever the weather condition is it affects you. You feel lethargic, you have no energy and you cough. It’s not an easy disease to get along with.

Interviewer: so how do you get along with it?

Patient: I carry on how I am you know, I except what I have got and I adjust in to it.

Interviewer: So you said you built up information as you went. What sort of information would have been useful at the start when you were diagnosed? Not just information, what else could have been offered to you?

Patient: I think there is more awareness now, I don’t think there was a great deal available at the time. So I can’t see it that way. As I said I was surprised to see how many people got the problem.

Interviewer: so when you meet with other people, what’s it like at the support group?

Patient: we have a cup of tea, cake and we talk about how things are. They get people from different ways of life to talk about the problem. Rest of the conversation was deleted because it involved a political figure and is not relevant). There was a lady from wales health and she talked about the problem and what is happening. We had a talk about what benefits we are entitled to. It’s just people getting to gather and talk.

Interviewer: what do you thing the biggest part?

Patient: just having a chat about families and how they are coping, you know that’s the biggest part of it. Getting to gather and talking to people with the same problem

Interviewer: so it’s a positive thing?

Patient: Oh yes.

Interviewer: do you experience any negative result?

Patient: No, not really. I'm a half glass full person.

Interviewer: what kind of industries have you worked in the past?

Patient: I worked in the steel industry. I worked in templates section. I had to go to the plant quite a bit, talk to people out there so if that had to do with anything I don't know. I used to work in an old building with asbestos walls, sometimes when you move a chair to the wall and the asbestos sheet cracks and if it's a part of the problem, I don't know. The house I am living in has plaster ceilings and it had black motor (type of a dust) and I don't know if it caused the problem.

Interviewer: going back to the rehab programme, how did you find that?

Patient: very good. It was on Tuesday and Friday, 1st hour was exercises and the second hour was a cup of coffee and talk about the illness.

Interviewer: the exercises you did, was it weight based, was it standing, seated?

Patient: there was a treadmill, a rowing machine and a couple of bicycles which I didn't like. The weights the medicine ball things, the stretch band, leg weights, so there was plenty there. But you did what you wanted alternating between arms and legs.

Interviewer: what did you like about it?

Patient: again it was the people with the same problem, finding about how they coped. (Deleted from the transcript because the patient was talking about another patient in the rehabilitation programme)

Interviewer: did you enjoy the exercise part of it?

Patient: yes. Some of them were hard work. I mean the treadmill is fine, I was happy with that but it's hard to do with the oxygen on.

Interviewer: what kind of benefits did you feel from that?

Patient: it was nice to see people, it became a close gathering eventually. At the end we were missing each other. It was about talking the problem with the people who are aware about it. When I talk to people from work they have no idea what they are talking about, because they don't have a clue.

Interviewer: so you are looking forward to the next one?

Patient: yes.

Interviewer: what would encourage you to go to an exercise class?

Patient: I want to go back. To be honest o don't need encouragement. But I was going to a private gym but I don't know if they would allow me to use oxygen because it is a private gym. But the local gym the trainers have been trained to handle oxygen and aware of it so I feel safer.

Interviewer: are there any exercises that you would prefer to do?

Patient: the treadmill. I walk uphill on it. I go on it for 25 minutes and I go on weights for 25 minutes and that will be it then.

Interviewer: there are some people in your situation that don't want to do exercises. What do you think that will encourage them to start doing exercises?

Patient: I think lot of them have depression. I don't know if it is the medication or how they are. They have a different look of life.

Interviewer: what does having IPF mean to you?

Patient: I accept the illness. I can't avoid the end of it because I know what is coming. But I make the most of what I got with whatever the time I have got left. It's a condition which affect all ages and areas of life. (Rest of the conversation was removed because the patient was talking about other patients involved in the rehabilitation programme).

Interviewer: what does your family think about your diagnosis?

Patient: my daughter worries, but my son had a shock when he saw me with the oxygen. He didn't realize how bad it was. (Rest of the conversation was deleted because the patient was talking about personal matters relating to his son).

Interviewer: are they supportive of you being active and doing exercise?

Patient: I got told off by my daughter cutting the grass. But I got to do it. I got to do it when I get the chance.

Interviewer: what do they think about the exercise classes?

Patient: they think it's quite good. And the grandchildren cares a lot. They encourage me to go. I think it does me good.

Interviewer: is there anything you would like to add?

Patient: I think when I was diagnosed little was known about the illness. So it's only in the past 5-6 years that they have known more about it. If I can help in anyways I try to do it.

END OF THE TRANSCRIPT

PATIENT INTERVIEW TRANSCRIPTION

(patient 7)

Age: 53

Conducted by: JH, SS

START OF THE TRANSCRIPT

Interviewer: 1st thing which will be really useful for us is to get an idea of the context of IPF, when you were 1st diagnosed with IPF.

Patient: First I was having all this trouble last year and I had a medical problem from that I went to get sort it out and they took a scan and dealt with it. The doctor said besides that “I had a look at your lungs and I’m not happy with it and I’d like to refer you”, and I thought nothing of it to be honest, I thought ya fine no problem.

Two months down the line I got referred to a guy called Dr. []. He gave me a few tests to take and said he thinks it IPF, but we need to diagnose it to see what it was. So I had a biopsy in January or February and it came out to be what they thought it was. He said because of my age I could be a good candidate for a new set of lungs but he had to take it to the committee and put my name forward. So at the moment I’m in limbo. There is this new medication which stops your lungs repairing itself but you have to be in a certain percentage. At the moment I’m in the good side rather than the bad side and what will happen is, when I drop to the bad side I start taking the medication and that’s when I will be there and the next line. And if I don’t get a lung transferred at that point and they will say it’s too late and that’s where I am at the moment. So flight of stairs panting like a bulldog. Anything uphill panting like a bulldog. It’s horrible. If I bend down quickly I feel dizzy. It’s been really restrictive what I want to do in the house, garden, if I carry anything heavy its too much, things like that. It’s a nightmare.

Interviewer: so it’s quite resent for you?

Patient: ya, quite resent. After operation I went back to work and they made major changes to my job to make it easier for me. It been really good work wise, they encourage me to work because if you push the lung its better because if you sit and work your lungs will go bad faster. So that’s the position I’m in now.

Interviewer: what do you do for work?

Patient: I’m a refuse carrier and walking 15-20 miles a day picking up rubbish, I couldn’t do that anymore. So they put me on to sweeping side where they gave me a van where I go around the shops and do the bins. That what I do which is a lot easier now. So I’m walking 20-30 yards maximum back in the van go to the next one.

Interviewer: is that what you have always done?

Patient: I have done that for the last 25 years. Before that I was delivering bread and before that I worked for Sainsbury.

Interviewer: was that doing manual work?

Patient: yes.

Interviewer: in a factory?

Patient: in a supermarket running produce and frozen foods.

Interviewer: have you ever been a smoker?

Patient: was a smoker, a 20 man a day up till the age of 26. As soon as we had our 1st daughter I stopped. Don't drink don't smoke.

Interviewer: How old are you

Patient: I am 53 now.

Interviewer: being diagnose with IPF last year, how did you feel about that?

Patient: pretty depressing, I've got to be honest, I've look it up on the internet got some scary bits and told my wife there are some scary things out there that I don't want to know about, so if we are going anywhere you are going to come with me and when they we start talking about horrible things I'm gona get up and walk out because I don't want to be worrying about it. So she has taken all of that. I think I've got 5 years if I don't get a set of new lungs and on medication it may extend it to ten years. But when it going to go to that stage and have to carry a bottle around like that I don't know. It's pretty scary. Because what I was doing, with all the rubbish I was picking up, I thought ill blame the council, and when they did the biopsy they didn't find anything. Now they have all the health and safety rules, totally different ballgame. Couple of years ago I was picking up stuff where people come and dump thigs off vans and it was building stuff like plater board, so I think that could have caused it as well. But I can't prove it so I have no case against the council.

Interviewer: it a difficult condition isn't it

Patient. Yes, sitting here I look fine. They say there is nothing wrong with you, try walking flight of stairs it's really horrible.

Interviewer: what kind of information did you get? Did you get right information, lot of information?

Patient: No, my wife saw what the doctor was writing and she got some letters off it and looked it up 1st to see what we were getting in to, and it's nasty. There are things that I don't wana know down the line. She knows I don't, I would rather keep it that way.

Interviewer: sounds like she is great support.

Patient ya, she is. Really good support.

Interviewer: you also mentioned some advice that you got about keeping the lungs moving

Patient: they offered me a course at [] for 7 weeks where you go twice a week for an hour, basically it was doing different exercises. I thought with the work I do I don't need to do exercises. I got a dog and I walk with him up and down a canal and get quite a distance. At the end of that term my improvement worked out to be 33% in my lung capacity. I was doing rowing machine, steps, treadmill and I was really getting breathless but I went really hard. May be there is something in it.

Interviewer: what type of exercise would you like to do?

Patient: I could probably do biking, walking, running is out, that's about it actually, but if I am going out I need to plan a route which I know is flat. I want to get somewhere I want to get back and if I can't get back I want to call somebody to ask come and pick me up. I feel like an old man in 90. I think what's going on here, it's not right you know

Interviewer: given this diagnosis, what does it mean to you to be diagnosed with IPF?

Patient: I feel picked on, I feel I haven't smoked for all these years, I don't drink, I'm not overly fat, why is this happening to me? There are lot of people who are older than me who haven't got it. My father died when I was very young so I don't know if he had it, my mother didn't have it, if it a trait in the family, I don't know. My brother is OK, so it looks like I'm the 1st to have it in the family. It's not good but you would have to get on with it.

Interviewer: obviously there are lot of negatives associated with the conditions, do you see any positives?

Patient: positives would be my age, I'm a good candidate for a lung transplant.

Interviewer: are there any kind of stipulations about your suitability

Patient: No. It's just a case of how bad it gets. At the moment I'm too fit to have them, it wouldn't benefit me. It's a fine line. If you go below where you are not fit enough to handle it they won't do it either. That where I don't want to get to. That's my main fear.

Interviewer: going back to the exercise programme you did, you said it improved your lung function. Were there any other benefits from it?

Patient: I'm sleeping better on the days I was doing it. Social thing was OK, it's nice to say, it's a horrible thing to say, but it was nice to see there were people worse than I was. Because lot of people were carrying the bottles around and they had tubes up there nose and they were hardly doing anything. And I told myself I am not that bad yet. That was a bonus. I also had few good discussion about what happens, what you get, because there are so many different varieties of it. And I learnt few things like when it comes to climbing steps it's easier to breathe in when u take off the step and breath out when you land on the step. Ya little things like that.

Interviewer: would there be more things like that, which would be useful do you think?

Patient: don't know

Interviewer: What types of exercises do you think that will be useful in an organised programme?

Patient: doing weight was quite useful because that's all breathing as well. Treadmill was OK, because you could go on speeds you want to go. Rowing was a bit hard because it crunches and make it hard to breath.

Interviewer: you mentioned things that are good to do and not good to do, and the reasons, are there any others like rowing?

Patient: rowing is not good, we did cross trainers and it was a killer because you use so many limbs at the same time. I tried doing that and in 3-4 minutes I was done for the day.

Interviewer: are there any others that are useful? Any daily activities?

Patient: bending thangs up is a problems, carrying things is a problem, heavy weights area a problem.

Interviewer: what do you think motivates people to go and take part in exercise training programmes?

Patient: if thy carried on what I was doing with this twice a week I would have carried on doing it. It was disappointing that it ran for a limited time. Because you met people and after that you got a cup of tea sit down and had a chat. It was nice. But you won't get that in a gym.

Interviewer: does your wife encourage or discourage you to exercise?

Patient: she can go both ways. If she see me doing something I shouldn't do, she would ask the girls to do it. Other than that if I say I need to do it she would let me. If I go up the stairs and I forget something she would ask the girls to do it and I would say I will want to do it because I need to do it. Some. Eventually she is talking about us moving downstairs and stay downstairs once it gets to a certain point, well I don't want that. I'd rather take 10 minutes to go upstairs to go to bed than staying down stairs all the time. That's just me

Interviewer: so you are trying to stay active in your day to day life style?

Patient: ya, I'm not pushing it too much but I want to push it to a point where you start getting breathless.

Interviewer: about the exercise programme, are there any other important elements that you thing relevant?

Patient: there were lot of attitude, mentally how you look at it. Don't look at it half empty and but half full. I suppose in a way that does help.

Interviewer: was that a structured session?

Patient: no it's just patients talking while exercising. Its informal thing.

Interviewer: is there any other thing that you would add to it?

Patient: I would make it a bit longer. The individual session. Because there was a warmup and you would lose 20 minutes and it was a lot of time lost. I like to see that extended for a couple of hours.

Interviewer: is there anything more to add?

Patient: no I don't think so. There are not lot of information out there and there are many varieties of it. So what's good for me might not be good for someone else. And that's a big things. And what information out there it's scary. They say what you have and what will happen in the end and that's it. There are no information on what will happen in between.

Interviewer: if you were to tailor how information would look like, is there a better way to present it?

Patient: I think you need it in age groups, which would help. When I went to the doctor he said if you were 20 years older I would say you can't do anything, but since you are such a young age you have options. So you need it in age groups. If you have that my route will be there to there to there and in 10 years it will be there to there.

Interviewer: thank you for taking part in the study.

END OF THE TRANSCRIPT

PATIENT INTERVIEW TRANSCRIPTION

(patient 8)

Age: 73

Conducted by: JH, SS

START OF THE TRANSCRIPT

Interviewer: when did you get your diagnosis on IPF?

Patient: it was about 12 months ago now, very shocked, nearly upset at the time. Coming to terms with it slowly but that's life. I was please it wasn't cancer. Because I wasn't able to stop coughing at that time.

Interviewer: so the shock was about your life style?

Patient: well no, I just had this cough, it went on and on so I went to the doctor. I also get this pain in my chest. She send me for an asthma test and it was negative. Then a heart scan and it was OK. And I was still coughing I thought I have cancer. Then she sent me to Dr [] and I had all these test. After that I met his registrar. He went in met Dr. [] and he came out and I though oh god it cancer and he said you have got a lung disorder. But I want to discuss it with my colleagues before I diagnose what it is. And after my holidays I came back and he said you have fibrosis in the lung. I though thank god it's not cancer but I was upset at the same time. They gave me a booklet and I wish I didn't read it. It said I would life for 3 years but I've since heard that people live for 7-10 years and I calm down a bit. That came as a great shock. I used to be a smoker but they said that day don't know that smoking caused it. (Rest of the answer was removed because it wasn't relent to the topic)

Interviewer: what do you do for living?

Patient: I've done all sort of things. I was a supervisor at CNA's for a year. I was on the management programme. Then I worked in a solicitor's office, then I was a receptionist at a doctors before I retired.

Interviewer: but no kind of heavy industry?

Patient: no, never, nothing like that. I've had a stressful life, don't know if that had anything to it.

Interviewer: at the time did you get enough information or right kind of information on IPF?

Patient: to give it to someone as soon as diagnosed it, I think it was not a good idea. I read it and saw three years I though oh my god. Then my daughter read it and she was badly distress and she kept it from me. When it sitting there in front you think OMG.

Interviewer: you said you have come to terms with it.

Patient yes I have started to come in terms with it. These days I think oh god what is going to happen to me, am I going to end up in a nursing home or dead and my daughter says you will never go in to a nursing home but you know these days you can't say that, you never know what's going to happen. My husband is not well too. And if I get up thing I get more out of breath.

Interviewer: have you got any way of coping it?

Patient: well to be honest I carry on and tell myself I live another 20 years and I get on with it. That's the only way I could cope with it.

Interviewer: how old are you?

Patient: 73

Interviewer: on a day to day basis how does your condition affect you?

Patient: I find it very difficult going upstairs, I get out of breath. I'm overweight and have knee replacement and that doesn't help. But my daughter help me change the beds because I find that difficult. And if I walk a lot I get out of breath. But other than that I go out and have a nice time. I just came back from Spain last month and I was a different, the weather was so nice and the moment I came back it was so bad. I was veezing and it was so bad. The weather makes a difference without a doubt.

Interviewer: Although you get out of breath, do you do what you normally do?

Patient: Oh no. I used to play golf and I can't do that anymore. I can't dance. I play bridge a lot and that's my saviour. Other things I manage to do. Like walking the dog on the flat.

Interviewer: how often do you walk the dog?

Patient: I don't do it mush these days because they are with my daughter, but when we have the dogs it at least three time a week.

Interviewer: does anybody in the hospital talked about any exercise or rehab programme?

Patient: no. I've been on the rehab programme. I enjoyed it. I thought it was good. (The rest of the conversation was removed because she was talking about other patients). I think if you are going somewhere (rehabilitation programme) the people you are going to should know about the illness and should know how far you could go. I'm waiting to go to one of the programs and I don't know what it's like. The nurses who is sorting this out should check what they do before they push you out. But I think it was good, the one in the hospital.

Interviewer: what was good about the hospital one?

Patient: I felt safe. There were people there watching you and when you had enough you could stop. There was company, people with the same disease. I found I was looking forward to going. And I thought it was doing me good.

Interviewer: did it help you physically?

Patient: ya it was. Can't put a finger on it, but I feel better in myself. I also lost half a stone.

Interviewer: did you feel any better in any other way?

Patient: not really no. talks afterwards, I found it stressful. I didn't find it useful because I wasn't able to cope with that, the stress I have gone through. The breathing exercise was good. There was a lot of good. The actual gym I enjoyed.

Interviewer: dis you find the exercises they had useful?

Patient: yes. They had the walking machine, the bike. They had starch bands. And I found them help in some ways.

Interviewer: what type of exercise would you like to see in the programme in panarea?

Patient: the bicycle, the walking machine. I like the elastic band. The medicine ball. Weights. I found them very helpful.

Interviewer: did you manage to do weight standing?

Patient: yes. (The rest of the conversation was removed due to irrelevance)

Interviewer: was there anything that you would add to it?

Patient: no I don't think so.

Interviewer: what's it like to your family (the disease and exercise)?

Patient: my daughter knows, my husband knows. He had been very protective in a loving way. My son, haven't told him.

Interviewer: what does your husband and daughter think about you exercising?

Patient: they want me to do it. My husband think it's good. He wants to go on walks and then I worry about him because of his situation. But we do go for walks.

Interviewer: do you think the time duration of the individual workout session was enough.

Patient. Yes. May be 15 minutes more on the exercise part would have been better and cut down the talking session. And I would like to carry on the programme for more than six week.

Interviewer: what are your thoughts about the support group?

Patient: I like going there. They have people coming in and talk about the illness. Dr [] is there all the time. And it nice to chat with people with the same problem. When I see people with oxygen that what I dread. I don't know if I could cope with it. If you want to live you live but it's not me. (Rest of the conversation was deleted on patient's request).

Interviewer: what does having IPF mean to you?

Patient: Death. Certain definite death. That's what it means to me.

Interviewer: is there anything positive that come out of your diagnosis.

Patient: no, unfortunately no.

Interviewer: were you able to find information on IPF easily?

Patient: yes I got that booklet. Even without that there are lot of thing in the internet.

Interviewer: thank you for taking part in the study.

END OF THE TRANSCRIPT

PATIENT INTERVIEW TRANSCRIPTION

(patient 9)

Age: 65

Conducted by: JH, SS

START OF THE TRANSCRIPT

Interviewer: When were you 1st diagnosed with IPF?

Patient: 1st time I realized that something was wrong. I entered this cycling competition with my son that was a year ago. There were couple of young boys cycling around us. These boys accelerated and I thought I can do that and so I went couple of yards and I felt something was wrong. So I took some rest on the pavement. My son told let's stop and I don't like giving in so I walked rest of the way. I took some rest, had a banana and I was completely fine. And that was the beginning of it. (Rest of the conversation was deleted due to irrelevance).

Interviewer: so it sounds like you were very fit back in the day?

Wife: you were when you were in the army because you had to do all those exercises and walk everywhere.

Patient: Yes. I used to walk on every Friday to see my girlfriend.

Wife: you haven't been too bad have you?

Patient: when I get it its not very pleasant. I get breathless, can't talk properly, my voice goes for some strange reason and it's not very good. But I live

Wife: He rides the bicycle indoors so that's good. Sometimes he is not very good but he is ok.

Interviewer: so everyday life hasn't changed a lot?

Wife: not really. He does get quite breathless. What it does really is that he can't walk too far. Also his throat goes. I think there is a problem with his throat as well. Sometimes he can't speak. We went on a trip to Australia and new Zeeland and the before the trip he said he can't go. But after going to the doctor we decided to go. It affects him a lot but it doesn't stop him doing what we want to do. So it's been there for a long time but he had been able to cope with it.

Interviewer: so how have you coped with it for such a long time?

Patient: I've been on the exercise bike; I've been walking slowly and doing gentle exercise. So I have been taking care of myself.

Wife: he is taking care of himself so he is controlling it by not straining himself too much.

Interviewer: is it because you had advised on it when you were diagnosed?

Patient: he is being seeing Dr. [] only for couple of years.

(Rest of the conversation was deleted from the conversation because it was irrelevant to the topic)

Interviewer: When you met Dr. [] did you get a diagnosis on your breathlessness?

Patient: I saw a young doctor 1st and she discovered my lungs were scared. Ten only I was diagnosed with IPF. I used to be a heavy smoker.

Interviewer: so when you were diagnosed how did you feel about it?

Patient: it came as a little bit of a shock. It affected me but not in an excessive way.

Wife: sometimes you get very breathless and I notice when that happens he is very tired. (Rest of the conversation was removed from the transcript because it was irrelevant to the topic.

Interviewer: if we were to offer you an exercise programme what would you like it to be?

Patient: as long as it's not strenuous I can do it. (Rest of the conversation was removed from the transcript because it was irrelevant to the topic)

Interviewer: So what kind of exercises do you think you could do?

Patient: I can certainly go on the exercise bike, nothing hard but I find it useful for circulation on the legs.

Interviewer: do you share everything about your disease with each other?

Patient: we are pretty open about it and it been an excellent marriage. (Rest of the conversation was deleted from the transcript because it was irrelevant to the topic)

Interviewer: when you 1st found about your condition did you get enough information about it?

Patient: the impression that I got was that they haven't reached to a stage in the repair process of the disease and this is what's it's all about. They are trying to get more information.

Interviewer: have you got your own information about how might exercise help or not?

Patient: well, I find that the stretching is good and the exercise bicycle is wonderful. We also go for walks around the lake.

Interviewer: what is it about stretching that you find useful?

Patient: my intercostal muscles were very stiff, so I wasn't able to breathe properly. So I stretch and eventually I get a comfort through that. And the more I did the better it became. (Rest of the conversation was deleted because it was irrelevant to the topic)

Interviewer: you mentioned about stretching. If you were asked to create a rehabilitation programme to manage your IPF, what would that look like?

Patient: starching is really good and individual would be better because I can stop and start whenever I want and it would help others too.

Interviewer: finally, we have technology everywhere. Do you think technology could help you with exercise?

Patient: no it's not for us.

Interviewer: Thank you for taking part in our study.

END OF TRANSCRIPT

PATIENT INTERVIEW TRANSCRIPTION

(patient 10)

Age: 60

Conducted by: JH, SS

START OF THE TRANSCRIPT

Interviewer: When you got your diagnosis what sort of information did you get?

Patient: my background is in nickel and I have been exposed to obnoxious chemicals dust and so on. When I was 1st diagnosed I had a persisting cough like most people and it didn't clear for months. So I went to see a consultant and they put me through a cat scan and that's where they picked it up.

Wife: but he had been having the cough two years before that.

Interviewer: how did you feel when you 1st got your diagnosis?

Patient: Its ok. I didn't know anything about it and I've been living for 10 years.

Wife: we found lot of information on the internet and it was a bit devastating because he is quite healthy. But we got on with life.

Patient: lot of people don't know what it is. They understand lung cancer but when it comes to IPF they are like what is that? (rest of the conversation was deleted because it was irrelevant to the topic.)

Patient: also I've been on nine drugs which I didn't like at all.

Interviewer: Tell me little bit about the psychological effect of your diagnosis?

Patient: well, I wasn't happy on 9 tablets a day. It also getting grips with this condition. You have your ups and downs. It's a very debilitating condition. You have bursts of energy and bursts of you can't do anything. Simple things like taking out the bins, moving the lawn, cutting the bush such is very frustrating being used to carry out these things. But you have to accept it and carry on. It's very frustrating.

Interviewer: But you accept it?

Patient: you have to. I mean the fatigue you get when you are doing these things, I feel like a battery, your energy levels go down and you would have to stop. With 6 grandchildren we had a busy summer. During the holidays they were with us and I wasn't able to involved with them as much as I liked.

Wife: I think this new tablet he is on has increased his energy levels and psychological effect is good.

Patient: but it's been a little stressful taking care of the kids. (rest of the conversation was removed because it was irrelevant to the topic).

Interviewer: did you find your stress made your condition worse?

Patient: not really. I don't like it when stress affects me.

Wife: we get on with it. Stress id part of life isn't it.

Patient: 40 years in the chemical industry I've had disasters and stress but at the end you accept it and move on with life.

Interviewer: it sounds like that how you have come to terms with IPF.

Patient: you have to try to make the best of life. That's the way the dice is rolled. I do get frustrated when its physical affects me, not being able to do things that I planned to do. You think about retirement and think it's nice not to go to work but the health doesn't keep up. You plan to do all these things that you wanted to do all these years. But here we are.

Wife: we did them as much as we could. We travelled a lot, but the past three years not so much.

Patient: I've also been on the rehabilitation programme at [] and its really good. You do half hour of exercise and the we had lectures on the condition and it was really helpful.

Wife: the social side of it is really good. You share ideas and share how things are and we pass ideas.

Interviewer: so it gives you a support mechanism?

Patient: yes, you get to see the different stages of the disease and lot of guys there use oxygen. I have oxygen but I only try to use it in an emergency.

Interviewer: how did you find the exercise part of the programme?

Patient: good. I've always exercised and I'm still a member of LC2. Next week we will start going back again. It benefits physically as well as mentally.

Wife: we have friends coming in as well. So it really good.

Interviewer: the mental and the physical benefits of your exercises, could you explain it a bit more?

Patient: I have always exercised. When this condition came along, you start to worry about if exercise good or bad for you. I found after exercising I slept better, woke up better and it improved my flexibility. Mentally you are feeling better too and it helps. And with more exercise and steroids I'm desperately trying to put some weight on and improve my condition.

Interviewer: what kind of exercise do you do?

Patient: I can go on the walking machine. I can do three kilometres at 6kmph in 30 minutes. I do rowing for 10 minutes. Then I do pull ups with weights and some machine exercises. At the end I do spend about one hour there.

Wife: before the summer holidays there is a big improvement in his attitude. He is positive and I think it's the new drugs se is taking. During the winter I was worrying that he would go for depression. But it had made a big difference. (rest of the conversation was deleted due to irrelevance to the subject).

Interviewer: you said during the winter your mood was down. Do you think exercise helped you improve apart from drugs?

Patient: Oh yes, definitely. We will get back to it after the summer holidays.

Interviewer: going back on the exercise programme, you said you did a lot of walking. So did you do the rest while seated?

Patient: most of it was seated. And most of them were strength once.

Interviewer: if you were to go on an advanced rehab programme, what should it look like to you?

Patient: I think the course at [] is good. I could cope very well. It didn't tax me that much.

Wife: we have always done a lot of walking.

Patient: I'm ok with flat but with angles it hard.

Interviewer: staying active, is it hard to do?

Patient: no. you just have to change yourself. We are going to start the gym again.

Interviewer: if you were asked to exercise at home as a part of a rehabilitation programme. Is it something that you would follow.

Patient: They did it. I can't say I picked it up. It easier for me to jump in the car and go to the gym. If you are in the house it very distracting.

Interviewer: how important is it for you to have somebody with the knowledge to be there with you when you are exercising?

Patient: not too important. I know what I've got and I choose exercised that I can do and can get away with.

Interviewer: do you think the individual session length of the exercise session at [] adequate?

Patient: I think half an hour is enough for most people in it.

Interviewer: Do you have anything else to add?

Patient: not really I think we have said it all.

Interviewer: Thank you so much for both in taking part in the interview.

END OF TRANSCRIPT
